# Genome wide study of tardive dyskinesia in schizophrenia

**DOI:** 10.1038/s41398-021-01471-y

**Published:** 2021-06-08

**Authors:** Keane Lim, Max Lam, Clement Zai, Jenny Tay, Nina Karlsson, Smita N. Deshpande, B. K. Thelma, Norio Ozaki, Toshiya Inada, Kang Sim, Siow-Ann Chong, Todd Lencz, Jianjun Liu, Jimmy Lee

**Affiliations:** 1 Research Division, Institute of Mental Health, Singapore, Singapore; 2 Zucker Hillside Hospital, New York, NY USA; 3 Feinstein Institutes of Medical Research, New York, NY USA; 4 Tanenbaum Centre for Pharmacogenetics, Center for Addiction and Mental Health; Department of Psychiatry, Institute of Medical Science, Laboratory Medicine and Pathobiology, University of Toronto, Toronto, ON Canada; 5 Genome Institute of Singapore, Singapore, Singapore; 6 Centre of Excellence in Mental Health, ABVIMS & Dr Ram Manohar Lohia Hospital, New Delhi, India; 7 Department of Genetics, University of Delhi south campus, New Delhi, India; 8 Department of Psychiatry, Graduate School of Medicine, Nagoya University, Nagoya, Aichi Japan; 9 Department of Psychiatry and Psychobiology, Graduate School of Medicine, Nagoya University, Nagoya, Aichi Japan; 10 West Region, Institute of Mental Health, Singapore, Singapore; 11 Department of Psychosis, Institute of Mental Health, Singapore, Singapore; 12 Donald and Barbara Zucker School of Medicine at Hofstra/Northwell, New York, NY USA; 13 Yong Loo Lin School of Medicine, National University of Singapore, Singapore, Singapore; 14 Neuroscience and Mental Health, Lee Kong Chian School of Medicine, Nanyang Technological University, Singapore, Singapore

**Keywords:** Pharmacogenomics, Predictive markers

## Abstract

Tardive dyskinesia (TD) is a severe condition characterized by repetitive involuntary movement of orofacial regions and extremities. Patients treated with antipsychotics typically present with TD symptomatology. Here, we conducted the largest GWAS of TD to date, by meta-analyzing samples of East-Asian, European, and African American ancestry, followed by analyses of biological pathways and polygenic risk with related phenotypes. We identified a novel locus and three suggestive loci, implicating immune-related pathways. Through integrating *trans*-ethnic fine mapping, we identified putative credible causal variants for three of the loci. Post-hoc analysis revealed that SNPs harbored in *TNFRSF1B* and *CALCOCO1* independently conferred three-fold increase in TD risk, beyond clinical risk factors like Age of onset and Duration of illness to schizophrenia. Further work is necessary to replicate loci that are reported in the study and evaluate the polygenic architecture underlying TD.

## Introduction

Tardive Dyskinesia (TD) is a persistent and potentially debilitating involuntary movement disorder characterized by choreiform, athetoid, and or dystonic movements^1,2^. TD is largely caused by antipsychotic treatment and was first described in 1957^3^. Although commonly observed in patients with schizophrenia, TD can occur in individuals with other psychiatric disorders, as long as they have been similarly exposed to prolonged antipsychotic treatment. The prevalence of TD in schizophrenia is estimated to be between 15 and 30%, although rates of TD have reduced with prescription of second-generation antipsychotics^4–6^. Nevertheless, second-generation antipsychotics still carry with them a risk of developing TD, and TD remains a clinically relevant phenotype as it has been associated with more severe schizophrenia illness, cognitive impairments, lowered quality of life and increased mortality^7–10^.

The pathophysiology of TD is currently unknown. Theories pertaining to dopamine receptor hypersensitivity, serotonergic dysfunction, GABA insufficiency, and free radical damage have been put forth^2,11,12^. It is postulated that TD is related to the schizophrenia disease process; recent reports have linked basal ganglia (caudate nucleus) volume reductions to TD in schizophrenia; notably, these samples were on second-generation antipsychotic—suggesting the pathological process towards TD is beyond neurochemical properties^13^. As TD is potentially irreversible with no effective treatment, there needs to be an added emphasis on the prevention and identification of genetic risk factors. Several genes (e.g.-, *DRD2*, *DRD3*, *MnSOD*, *CYP2D6*, *GRIN2A*, and *GRIN2B*) have been implicated in candidate gene studies, but replication remains equivocal^14–18^. Genome-Wide Association Studies (GWAS) performed on the TD phenotype suggested that the GLI family zinc finger 2 (*GLI2*), heparan sulfate proteoglycan 2 (*HSPG2*), dipeptidyl-peptidase 6 (*DPP6*), and GABA pathway genes could putatively be considered susceptibility genes for TD^19–23^. Nonetheless, these studies are limited by small samples and require further replication. Here, we report the findings of the largest GWAS of TD to date. We identified a novel locus at 16q24.1 (*P* = 3.01 × 10^−8^) and three suggestive loci (1p36.22, 6q23.2, 12q13.13; *P* < 5 × 10^−7^) associated with TD.

## Methodology

Participants from the Singapore Clinical and Translational Research program in Singapore, and the Clinical Antipsychotic Trials of Intervention Effectiveness (CATIE)^24–26^ study were included in the current report. All participants met criteria for DSM-IV diagnosis for schizophrenia. Tardive dyskinesia was ascertained via the Abnormal Involuntary Movement Scale (AIMS)^27,28^. After quality control procedures, 1406 participants (280 with TD), and 6,291,020 SNPs remained. Linear Mixed Models GWA was performed via GEMMA^29^, and independent cohorts across the two studies were meta-analyzed via fixed effects inverse variance approach in METAL^30^. GWAS summary statistics were then subjected to functional annotation for GWAS identified loci^31^, eQTL lookups, pathway analysis^32^, and transcriptome-wide analysis^33^, which were conducted to characterize potential biological mechanisms underlying TD. Further fine-mapping analysis was also carried out to identify credible causal variants^34–36^. A series of polygenic risk score analyses were conducted to compare the genetic architecture of TD with related phenotypes^37^. We also carried out a series of post-hoc multivariate logistic regression analysis to investigate joint clinical and genetic factors that predict the emergence of TD. Finally, variants previously associated with TD were compared with the current GWAS results^11,18,22,23,38–46^. Detailed methodological approaches are further reported in the Supplementary Information included with the current report.

## Results

### Demographics and assessment of tardive dyskinesia

Demographics are reported in Table 1 and Supplementary Table 1. There were 71.1% males and 28.9% females with TD. There was no significant difference in gender proportion between individuals with TD and those without, $\chi^{2}$ (1, *n* = 1406) = 0.142, *P* = 0.706. There were significant differences in age, *t*(1404) = −14.06, *P* = 4.0 × 10^−42^, age of illness onset, *t*(392.7) = −3.06, *P* = 0.0024, duration of illness, *t*(1390) = −11.28, *P* = 2.76 × 10^−28^, and antipsychotic dose measured in CPZ equivalent, *t*(516.3) = 3.73, *P* = 0.002 between individuals with and without TD. These differences in demographics and clinical characteristics were further modelled using a polygenic risk score approach that allows us to examine genetic effects for TD alongside demographics effects; results are reported in subsequent sections.

**Table 1:** Characteristics of tardive dyskinesia versus non-tardive dyskinesia samples.

	TD samples (*N* = 280)	Non-TD samples (*N* = 1126)
Gender (male/female)	199/81	813/313
Age (years)	55.51 (11.38)	44.23 (12.17)
Age of illness onset (years)	28.71 (10.71)	26.55 (9.50)
Duration of Illness (years)	26.52 (12.55)	17.22 (12.23)
PANSS (score)		
Positive symptoms	12.90 (6.01)	13.51 (5.99)
Negative symptoms	15.42 (6.89)	15.69 (6.99)
General psychopathology	27.44 (10.11)	27.96 (10.12)
Total	55.76 (20.67)	57.17 (20.40)
Antipsychotics, *n (%)*		
Typical only	170 (60.7%)	487 (43.3%)
Atypical only	66 (23.6%)	394 (35.0%)
Typical + atypical	17 (6.1%)	93 (8.3%)
None	2 (0.7%)	7 (0.6%)
Daily CPZ equivalent dose, *mg* (SD)	494.7 (494.30)	637.16 (679.07)
Total aims score	11.09 (5.11)	1.19 (2.02)

### Genome-wide association of tardive dyskinesia

**Fig. 1:**
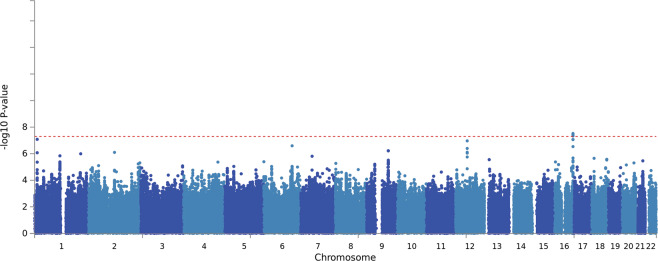
Manhattan plot for GWAS fixed effect meta-analysis of TD. Note: Three cohorts were included in the fixed-effect meta-analysis, STCRP, CATIE-EUR, and CATIE-AFR. A single GWAS signal was found at 16q24.1 for rs11639774.

**Fig. 2:**
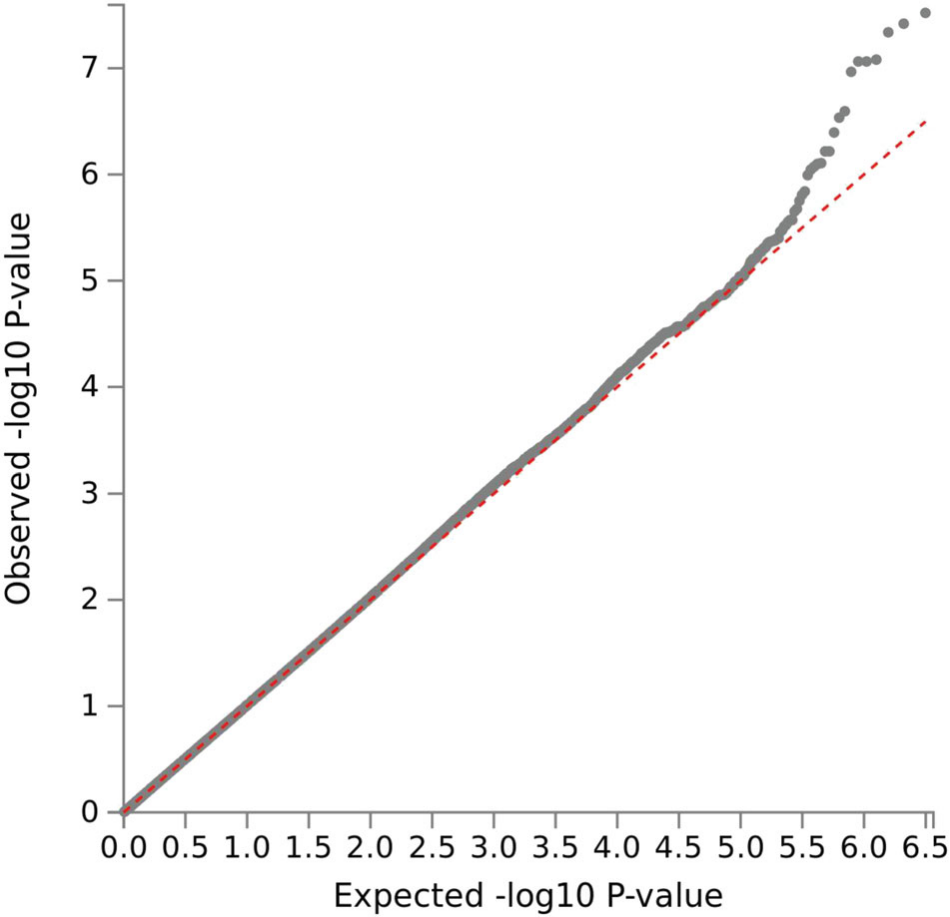
QQ plot for meta-analysis of STCRP, CATIE-EUR, and CATIE-AFR. Note: Lambda = 1.02.

**Fig. 3:**
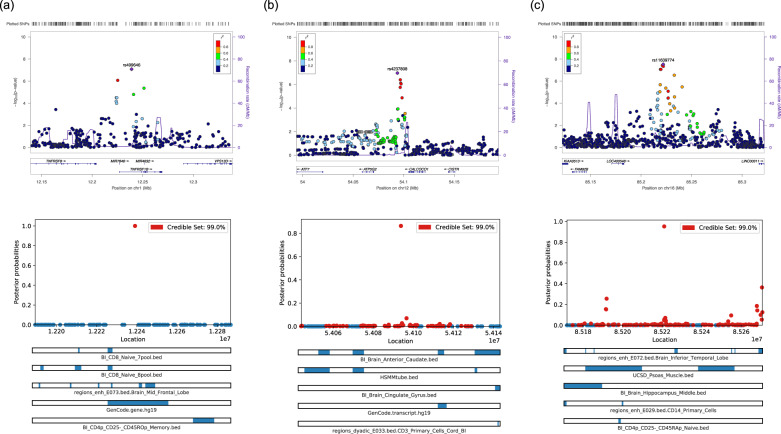
Regional loci and fine-mapping plots. Top panel represents regional plots for **a** Chromosome 1, **b** Chromosome 12, and **c** Chromosome 16. Bottom panel represents the visualization of 99% credible SNP set for **a** Chromosome 1, **b** Chromosome 12, and **c** Chromosome 16, against location of SNP, with top annotation bars.

Standard GWAS quality control procedures were carried out (Supplementary Figs. 1–5). We carried out Principal Components Analysis (PCA) on each ancestry group to identify overall population stratification across samples, and, within population PCA to identify fine-grain population outliers (See Supplementary Information). We removed population outliers detected by PCA via a series of *k*-means clustering. Notably, association analysis was carried out within each ancestry first and thereafter meta-analyzed. Linear mixed models conducted via the GEMMA^29^ package revealed significant genome-wide association of TD at the level of the CATIE cohorts, but not the STCRP cohort (Supplementary Fig. 6). Subsequent fixed-effect inverse variance meta-analysis^30^ (*λ*_GC_ = 1.02; Table 2, Figs. 1–3) across the STCRP and CATIE cohorts revealed one novel locus on chromosome 16 (rs11639774, downstream of *GSE1*) (*P* = 3.01 × 10^−8^). Three other suggestive independent genomic loci (*P* < 5 × 10^−7^) were identified on chromosome 1 (rs499646, *P* = 8.30 × 10^−8^, *TNFRSF1B*), chromosome 6 (rs6926250, *P* = 2.54 × 10^−7^, *EPB41L2*), and chromosome 12 (rs4237808, *P* = 1.08 × 10^−7^, *CALCOCO1*). Due to low minor allele frequencies in the STCRP sample, two of the SNPs were only present in the CATIE cohorts. Proxy SNPs in LD within the STCRP cohort were identified using the SNP Annotation and Proxy Search (SNAP)^47^. Further meta-analysis of association results from both proxy SNPs were carried out using Fisher’s *p*-value meta-analysis approach (https://CRAN.R-project.org/package=metap). Fisher’s meta-analysis for the top SNP (rs11639774) and proxy SNP (rs9928615) in the STCRP cohort was significant ($\chi^{2}$ (4) = 40.28, *P* = 3.79 × 10^−08^). Similarly, results for SNP (rs499626) and proxy SNP (rs11569835) in the STCRP cohort reached suggestive significance ($\chi^{2}$ (4) = 36.48, *P* = 2.31 × 10^−07^), with both primary variants with proxy SNP meta-analysis showing consistent effects (Supplementary Table 2). GWAS markers were further annotated via ANNOVAR, eQTL, Chromatin Interaction modules within the Functional Mapping and Annotation of Genome-Wide Association Studies (FUMA)^31^ (Supplementary Tables 3, 4; Supplementary Fig 7).

**Table 2:** Top SNPs associated with tardive dyskinesia.

SNP	Symbol	Location	Position	Variant	Effect allele	Other allele	EAF	Beta	SE	*P*-value	HetP
rs11639774	GSE1	16q24.1	85221868	Intergenic	A	G	0.168	0.151	0.0273	3.01E-08	0.067
rs499646	TNFRSF1B	1p36.22	12239089	Intronic	A	G	0.12	0.1949	0.0364	8.30E-08	0.246
rs4237808	CALCOCO1	12q13.13	54093797	Intergenic	T	C	0.4914	0.0765	0.0144	1.08E-07	0.506
rs6926250	EPB41L2	6q23.2	131302473	Intronic	T	C	0.6872	−0.0968	0.0188	2.54E-07	0.447

*SNP* single-nucleotide polymorphism, *EAF* effect allele frequency, *SE* standard error, *HetP* heterogenity *P*-value.

### Gene-based and pathway analysis

MAGMA was used to conduct both the gene-based and geneset analysis^32^. Gene-based analysis tests for association of TD with markers within the gene by mapping the SNPs to gene level, while geneset analysis aggregates individual genes to a collection of genes with overlapping characteristics^28^. Results of MAGMA gene-based analysis on 18,259 mapped autosomal genes after Bonferroni correction are presented in Supplementary Table 5. None of the genes were significant after Bonferroni correction. MAGMA competitive geneset analysis^32^ was conducted using the most recent Molecular Signature Database version 6.1^48^. Although none of the 17,199 genesets survived Bonferroni correction, top pathways implicated regulation of immunoglobulin production and immunoglobulin isotype switching, expression of chemokine receptors, and regulation of cell growth, and monocytes (Supplementary Table 6).

### eQTL lookups/transcriptome-wide analysis

Expression quantitative trait loci (eQTL) were performed as part of FUMA^31^ GWAS pipeline. Notably, eQTL effect of *ATP5G2* (Bonferroni corrected *P* = 1.24 × 10^−14^, Supplementary Table 7) and *MAP3K12* (Bonferroni corrected *p* = 6.29 × 10^−10^, Supplementary Table 7) was observed for subthreshold genomic significance loci for 12q13.13, *TNFRS1B* (Bonferroni corrected *p* = 4.60 × 10^−08^, Supplementary Table 7) for 1p36.22, *EPB41L2* (Bonferroni corrected *p* = 2.86 × 10^−07^, Supplementary Table 7) and *AKAP7* (Bonferroni corrected *p* = 1.48 × 10^−06^, Supplementary Table 7) for 6q23.2, with expression in various tissues, including those related to motor functions (Table 3).

**Table 3:** Summary of expression quantitative trait loci (eQTL) analysis.

Symbol	Chr	Tissue	Database
TNFRSF1B	1	BIOS_eQTL_geneLevel	BIOSQTL
AKAP7	6	BIOS_eQTL_geneLevel	BIOSQTL
AKAP7	6	Muscle_Skeletal	GTEx_v7
AKAP7	6	Thyroid	GTEx_v7
EPB41L2	6	xQTLServer_eQTLs	xQTLServer
EPB41L2	6	Skin_Sun_Exposed_Lower_leg	GTEx_v7
EPB41L2	6	Esophagus_Muscularis	GTEx_v7
EPB41L2	6	Esophagus_Mucosa	GTEx_v7
EPB41L2	6	Lung	GTEx_v7
ATF7	12	BIOS_eQTL_geneLevel	BIOSQTL
ATP5G2	12	Esophagus_Mucosa	GTEx_v7
ATP5G2	12	BIOS_eQTL_geneLevel	BIOSQTL
ATP5G2	12	Thyroid	GTEx_v7
ATP5G2	12	Nerve_Tibial	GTEx_v7
ATP5G2	12	Adipose_Subcutaneous	GTEx_v7
ATP5G2	12	Brain_Cerebellar_Hemisphere	GTEx_v7
ATP5G2	12	Brain_Cerebellum	GTEx_v7
ATP5G2	12	Testis	GTEx_v7
ATP5G2	12	Esophagus_Muscularis	GTEx_v7
ATP5G2	12	Adipose_Visceral_Omentum	GTEx_v7
ATP5G2	12	Small_Intestine_Terminal_Ileum	GTEx_v7
ATP5G2	12	Lung	GTEx_v7
ATP5G2	12	Skin_Not_Sun_Exposed_Suprapubic	GTEx_v7
ATP5G2	12	Artery_Tibial	GTEx_v7
ATP5G2	12	CRBL	BRAINEAC
ATP5G2	12	MEDU	BRAINEAC
ATP5G2	12	PUTM	BRAINEAC
ATP5G2	12	SNIG	BRAINEAC
ATP5G2	12	FCTX	BRAINEAC
ATP5G2	12	aveALL	BRAINEAC
ATP5G2	12	TCTX	BRAINEAC
ATP5G2	12	WHMT	BRAINEAC
ATP5G2	12	OCTX	BRAINEAC
ATP5G2	12	THAL	BRAINEAC
ATP5G2	12	HIPP	BRAINEAC
ESPL1	12	Esophagus_Mucosa	GTEx_v7
MAP3K12	12	BIOS_eQTL_geneLevel	BIOSQTL
NPFF	12	BIOS_eQTL_geneLevel	BIOSQTL
SP1	12	Esophagus_Mucosa	GTEx_v7
SP7	12	Thyroid	GTEx_v7
SP7	12	Testis	GTEx_v7

Transcriptome-wide analysis was implemented via MetaXcan^33^. Unlike eQTL lookups, the MetaXcan approach further incorporates evidence from GWAS summary statistics with genome-wide gene expression profiles from the GTex^49^ database. This provides information of potential functional enrichment within a genomic locus. Here, we found lower expression of a transcript, *EPB41L2*, in the esophagus muscularis at Bonferroni-corrected significance (*P* = 0.02, Supplementary Table 8).

### Fine-mapping analysis

Cross-Ancestry fine mapping was performed on PAINTOR v3.1^34,35^ to identify putative causal variants on the 4 loci (1p36.22, 6q23.2, 12q13.13, and 16q24.1). The 99% cumulative posterior probability identified a total of 231 putative credible causal SNPs across three loci (1p36.22, 12q13.13, and 16q24.1; Supplementary Table 9, Supplementary Fig. 8). From these, highly credible SNPs were defined as posterior probability > 0.8. This identified a putative causal variant for each locus: 1p36.22 (rs499646, posterior probability = 0.99), 12q13.13 (rs4237808, posterior probability = 0.863), and 16q24.1 (rs28468398, posterior probability = 0.952). Notably, for loci on 1p36.22 and 12q13.13, the index SNP identified in GWAS is also a putative causal SNP identified by PAINTOR (See Fig. 3). Further annotations via the Variant Effect Predictor^50^ revealed that (i) rs499646 is an intronic variant 3500 bp from the promotor of *TNFRSF1B* and is a known protein-coding variant and appears to be a loss-of-function intolerant variant, (ii) rs4237808 is an intergenic variant between *ATP5G2* and *CALCOCO1*, but lies within 1000 bp of two CTCF binding sites, and 3200 bp from a known promotor flank, and (iii) rs28468398 is a regulatory region variant, which lies within a transcription factor binding site on *CTC-786C10.1*/*GSE1* gene. Notably, additional joint finemap annotations revealed that all three variants were enriched by known brain level gene expression sites close by (See Fig. 3). GCTA-COJO^36^ revealed no further signals present in the loci beyond independent variants identified via LD clumping or fine mapping (Supplementary Fig. 9).

### Polygenic risk modelling of TD with other diseases and traits

Polygenic risk score modelling via PRSice2^37^ (Supplementary Fig. 10) revealed best polygenic association of TD with amyotrophic lateral Sclerosis (ALS), albeit only two PRS thresholds remained significant after Bonferroni correction (P_T_ = 0.5; P_T_ = 1.0). ALS PRS-based pathway analysis indicated “misfolded protein” as a top pathway shared between TD and ALS (Supplementary Fig. 11), although this remains a trend finding. We followed up on the possibility that there might be pleiotropic genetic effects of ALS and TD, given that both conditions implicated motor neurons. A post-hoc lookup of ALS variants and genes was carried out on GWAS catalog. We filtered GWAS SNPs previously reported to be associated with ALS based on *p* < 1e-6 and extracted corresponding mapped genes. These were then looked up in the MAGMA gene results reported earlier. We found that three previously known ALS genes, *FBXO15*, *FAM19A1*, and *NP5* genes were nominally associated with TD genes (TD Gene *P*-values: *FBXO15:* 0.0210, *FAM19A1*: 0.0356 and *NPS*: 0.0455). Cross-Trait polygenic risk scores were also conducted on other psychopathological or autoimmune traits—such as schizophrenia, bipolar disorder, Alzheimer’s disease, Parkinson’s disease, depression, rheumatoid arthritis, and Crohn’s disease but were not significant after multiple testing correction. It is, however, notable that aspect of the schizophrenia and rheumatoid arthritis did appear to be close to the multiple correction threshold for *P*_T_ < 0.05.

### Post-hoc clinical analysis: effects of nedication type and SNP effects on TD

**Fig. 4:**
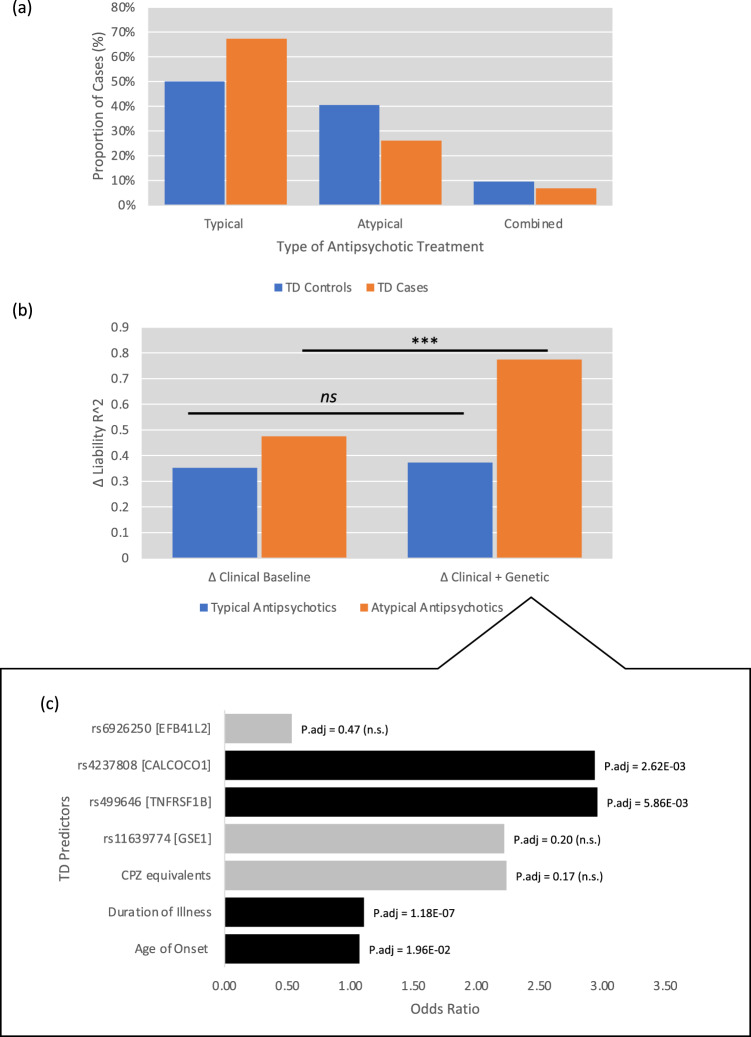
Clinical and genetic risk factors for TD. **a** Stratified analysis for antipsychotic type and TD **b** Logistic regression model for Clinical Baseline versus Clinical + Genetic Model. *Y*-axis: Liability scale R^2^ for logistic regression model, case-control proportions adjusted for TD prevalence set at 15%. Clinical Baseline: Age of Onset, Duration of Illness, Daily CPZ equivalents. Clinical + Genetic: includes Clinical Baseline + rs6926250, rs4237808, rs499646, rs11639774. ***p = 7.09e-8. **c** Odds ratio for predictors in Clinical + Genetic model. Black colored bars are significant after Bonferroni correction for all variables entered in the logistic regression model.

We performed post-hoc multivariate logistic regression analysis to investigate potential effects of clinical factors affecting TD beyond genetic effects. Preliminary linear-by-linear Chi-square analysis were significant ($\chi^{2}=18.67$, *p* = 1.40 × 10^−5^, see Fig. 4a). Expectedly, there was a significant proportion of TD cases taking typical, compared to atypical antipsychotics. The Clinical Baseline model (Age of Onset, Duration of illness, Daily CPZ equivalents) was significantly associated with TD, for individuals who were taking either typical (${\chi}^{2}=125.71$, df = 25, *p* = 2.15 × 10^−15^) or atypical antipsychotics ($\chi^{2}=75.57$, df = 25, *p* = 5.55 × 10^−7^). There were no significant model differences between the Clinical Baseline model nor Clinical + Genetic model (Clinical Baseline + rs6926250, rs4237808, rs499646, rs11639774) in predicting TD for individuals taking typical antipsychotics. However, for individuals taking atypical antipsychotics, there was a significant genetic contribution to predicting TD cases beyond clinical factors (See Fig. 4b, c, Δ$\chi^{2}$ = 38.96, df = 4, *p* = 7.09 × 10^−8^). It is notable that after adjusting for population stratification, database, and sex, Age of Onset (OR = 1.071, 95% CI = 1.029–1.114, p.adj = 1.96 × 10^−2^) and Duration of illness (OR = 1.105, 95% CI = 1.069–1.142, p.adj = 1.18 × 10^−7^) significantly predicted TD. Beyond clinical factors, two of the top SNPs rs499646 [*TNFRSF1B*] (OR = 2.957, 95% CI = 1.669–5.239, p.adj = 5.86 × 10^−3^) and rs4237808 [*CALCOCO1*] (OR = 2.937, 95% CI = 1.713–5.038, p.adj = 2.62 × 10^−3^) identified in the earlier GWAS significantly predicted the emergence of TD. Note that p.adj are Bonferroni adjusted *p*-values for 29 variables in the logistic prediction model (See Supplementary Information for further details).

### Lookup of past tardive dyskinesia studies

Variants previously found to be associated with TD were meta-analyzed with results from the current GWAS (Supplementary Table 10)^11,18,22,23,38–46^. The following variants, independent from CATIE were replicated in the current study *CYP1A2* (rs762551, *N* = 1725, *P* = 0.018), *DRD1* (rs4532, *N* = 1788, *P* = 0.045), *GRIN2A* (rs1345423, *N* = 1837, *P* = 0.012), *GRIN2B* (rs2192970, *N* = 1057, *P* = 0.0018), *HSPG2* (rs878949 (proxy), *N* = 1572, *P* = 0.0051), and *DPP6* (rs6977820, *N* = 1701, *P* = 0.00068). These genes implicated pathways involving drug metabolism, dopamine, and glutamate.

## Discussion

To our knowledge, here we report the largest GWAS for TD. A single novel locus at 16q24.1 was found to be associated with TD. We also identified three loci (1p36.22, 6q23.2, and 12q13.13) with suggestive evidence of association to TD. Of these, putative causal variants were identified for three of the loci. The top GWAS hit at 16q24.1, in the *GSE1* coiled-coil protein gene, encodes for a proline rich protein which was reported to be a subunit of *BRAF35*-*HDAC* (BHC) histone deacetylase complex^51^. This gene has been known to be implicated in the proliferation, migration, and invasion of breast cancer cells^52^. A lookup in the GWAS catalog revealed *GSE1* was also associated at GWAS significance with platelet count and distribution^53^, and suggestive GWAS significance with amyotrophic lateral sclerosis^54^ and sulfasalazine-induced agranulocytosis^55^. *GSE1* contributes to a geneset that are predicted targets of a microRNA biomarker for schizophrenia^56^.

Other suggestive associations with TD included *TNFRSF1B* (1p36.22), *EPB41L2* (6q23.2), and *CALCOCO1* (12q13.13). The tumor necrosis factor receptor superfamily member 1B (*TNFRSF1B*) is a protein-coding gene that mediates antiapoptotic signaling. Expression of *TNFRSF1B* is specific to cells in the immune systems, specific neuronal subtypes, certain T-cells subtypes, and endothelial cells^57^. This appears to be supported by results from the competitive geneset enrichment analysis, albeit nonsignificant, revealed top pathways that implicated immunoglobulin production, chemokine receptors, and monocytes. These findings appear to support existing theories on the role of immune and inflammation in the pathogenesis of TD^11,12^.

The erythrocyte membrane protein band 4.1 like 2 (*EPB41L2*) is involved in actin and cytoskeletal protein binding. More recently, *EPB41L2* deficiency was found to result in myelination abnormalities in the peripheral nervous system, leading to motor neuropathy in a mice study^58^. This putative association of *EPB41L2* deficiency is consistent with the direction of effect found in our GWAS results, suggesting that the expression of *EPB41L2* might confer a protective effect against motor neuropathy. Functional enrichment in and around the *EPB41L2* based on the MetaXcan finding is intriguing, further research is needed to dissect the function of *EPB41L2* in TD pathophysiology.

The calcium binding and coiled-coil domain 1 (*CALCOCO1*) is a protein-coding gene that is involved in the activation of transcriptional activities of targets genes in the Wnt signaling pathway, neuronal receptor and aryl hydrocarbon receptor (AhR)^59^. Notably, one function of the AhR, a ligand-based transcription factor, is the regulation of transcriptional activity for drug metabolizing enzymes, including the family of cytochrome P450 (CYP) genes^60^. The family of CYP has been postulated to be a candidate for TD susceptibility^11^. Specifically, CYP enzymes, such as *CYP1A2*, *CYP2D6*, and *CYP3A4*, metabolize antipsychotics (e.g., clozapine, olanzapine, and haloperidol) and antidepressants (e.g., fluvoxamine)^60^. Meta-analysis of *CYP1A2* from past and current study revealed that this gene was significantly associated with TD at trend level (*P* < 0.05; Supplementary Table 10).

Follow-up post-hoc investigation showed that individuals that were on Typical antipsychotics were expectedly more likely to be a TD case. We also demonstrated that clinical factors such as Age of Onset and Duration of illness of schizophrenia also significantly predicted TD. However, beyond clinical factors, top SNPs within *CALCOCO1* and *TNFRSF1B* were responsible for accounting for nearly three times the risk of having TD and that joint clinical and genetic model accounted for greater than 75% of variance for individuals having TD and were on atypical antipsychotics. Nevertheless, further investigation is required to confirm the replicability and generalizability of the observed phenomenon.

Polygenic risk score analysis on traits with polygenic architecture revealed overlapping genetic polygenic risk of TD and ALS. The shared protein misfolding pathway between TD and ALS is consistent with findings from our MAGMA pathway analysis. Post-hoc lookups of known ALS genes that might be associated with TD revealed that an F-Box protein-encoding gene *FBXO15*, chemo/neurokine encoding gene *FAM19A1*, and neuropeptide encoding gene, *NPS*. Proteinopathies commonly implicated in neurodegenerative conditions have been proposed to also underlie TD^61,62^. Prior reports have implicated the accumulative role of proteinopathies in neurotoxicity, synaptic dysfunction, and its bidirectional effect with oxidative stress, neuroinflammation, and its consequence on the immune system^61,62^. We do note, however, that genetic overlap between TD and other seemingly related traits is generally weak, such as neurodegenerative conditions and autoimmune conditions. Nevertheless, Schizophrenia and Rheumatoid Arthritis were close to the multiple correction threshold. Future work in more powered samples may be carried out to investigate how the potential biological mechanisms for ALS, rheumatoid arthritis, and schizophrenia might be related to TD. We note that potential ancestry differences in LD patterns and allele frequencies of the target and training dataset could result in poorer polygenic prediction. The generally small pharmacogenomic datasets utilized in the context of this report, and the use of multiple reference panels (i.e., HapMap and 1000 genomes project) could have contributed to the variability of the results. Nevertheless, pharmacogenomic datasets such as those reported here continue to allow more clues to be shed regarding the potential biological mechanisms that underlie complex clinical effects that arise from the prescription of psychotropic medication.

Emergent results from the current report demonstrate the polygenic architecture of TD. While the current study remains underpowered for GWAS analysis (Supplementary Fig. 12), we present the largest GWAS of TD to date. The findings reported here suggest that multiple overlapping biological systems might contribute to the etiopathogenesis of the condition. Taken together, results suggest that TD is associated with significant proteinopathies, disrupted neuronal function within the brain and potentially including muscular innervation, existing in a background of inflammation. Further work is necessary to identify the cascade of biological disruptions and events that trigger TD symptomatology. Further work is needed to replicate findings in the current report and further unravel the biology of these risk variants and pathways identified.

## Web resources

FUMA, http://fuma.ctglab.nl/.

GAS Power Calculator, http://csg.sph.umich.edu/abecasis/cats/gas_power_calculator/index.html

GCTA, https://cnsgenomics.com/software/gcta/

GEMMA, http://www.xzlab.org/software.html

GWAS Catalog, https://www.ebi.ac.uk/gwas/

LD-HUB, http://ldsc.broadinstitute.org/

MAGMA, https://ctg.cncr.nl/software/magma

METAL, https://genome.sph.umich.edu/wiki/METAL_Documentation

MetaXcan, https://github.com/hakyimlab/MetaXcan

Michigan Imputation Server, https://imputationserver.sph.umich.edu/index.html

MSigDB, http://software.broadinstitute.org/gsea/msigdb

PAINTOR, https://github.com/gkichaev/PAINTOR_V3.0

PLINK1.9, https://www.cog-genomics.org/plink2

PRSice2, https://github.com/choishingwan/PRSice/wiki

PredictDB, http://predictdb.org/

R, https://www.r-project.org/

SNAP, http://www.broad.mit.edu/mpg/snap/

Variant Effect Predictor, https://www.ensembl.org/vep

## Supplementary information

Supplementary Information

Supplementary Tables
