# Supplementary material for: Genome wide study of tardive dyskinesia in schizophrenia
Source: Transl Psychiatry. 2021 Jun 8;11:351. doi: 10.1038/s41398-021-01471-y (PMC8187404; doi:10.1038/s41398-021-01471-y)
Supplement: Supplementary file 1 — Supplementary Information [file 41398_2021_1471_MOESM1_ESM.docx]

Supplementary Information

Contents

[1. Cohort Information 2](#_Toc65281719)

[2. Phenotype Ascertainment: Tardive Dyskinesia 2](#_Toc65281720)

[3. Genome-wide Genotyping, Quality Control, and Imputation 3](#_Toc65281721)

[4. Genome-wide Association Analysis and Meta-analysis 4](#_Toc65281722)

[5. Functional Annotation for GWAS 4](#_Toc65281723)

[6. Pathway Analysis 5](#_Toc65281724)

[7. Transcriptome-wide Analysis 5](#_Toc65281725)

[8. Fine-mapping Analysis 6](#_Toc65281726)

[9. Polygenic Risk Modelling of TD with Other Diseases and Traits 6](#_Toc65281727)

[11. Logistic Regression Modelling for Clinical and Genetic Factors Predicting TD 7](#_Toc65281728)

[10. Look-up of Past Tardive Dyskinesia Studies 7](#_Toc65281729)

[11. Supplementary Figures 8](#_Toc65281730)

[Supplementary Figure 1. Missing SNP filters. 8](#_Toc65281731)

[Supplementary Figure 2. Missing individual filters. 9](#_Toc65281732)

[Supplementary Figure 3. Minor Allele Frequencies and Hardy-Weinberg Equilibrium. 10](#_Toc65281733)

[Supplementary Figure 4. F-het coefficients and IBS/IBD filtering. 11](#_Toc65281734)

[Supplementary Figure 5. Principal components analysis for detection of population outliers. 12](#_Toc65281735)

[Supplementary Figure 6. Manhattan and QQ-plots for STCRP-EAS, CATIE-EUR and CATIE-AFR. 13](#_Toc65281736)

[Supplementary Figure 7. Chromatin Interaction plots for TD GWAS significant (p < 5e-8) and candidate (p < 5e-7) regions. 14](#_Toc65281737)

[Supplementary Figure 8. Trans-ethnic fine mapping 17](#_Toc65281738)

[Supplementary Figure 9. GCTA-COJO conditional analysis for top SNPs 18](#_Toc65281739)

[Supplementary Figure 10. Polygenic Risk Score results. 19](#_Toc65281740)

[Supplementary Figure 11. Pathway based polygenic risk score results. 20](#_Toc65281741)

[Supplementary Figure 12. Post-Hoc GWAS power calculation. 21](#_Toc65281742)

[References 22](#_Toc65281743)

# 1. Cohort Information

STCRP cohort

The final analysis included 780 (n_TD_ = 188, n_Non-TD_ = 592) individuals with schizophrenia, aged between 21 and 55 years, recruited as part of the Singapore Translational and Clinical Research in Psychosis program (STCRP) from year 2005 to 2008. Prior to quality control procedures, genotype and phenotype data was available for 1939 and 843 schizophrenia individuals respectively. Schizophrenia individuals were recruited from inpatient wards and outpatient clinics at the Institute of Mental Health, Singapore. All cases were of Han Chinese descent. Participants with a history of neurological injuries, mental retardation and substance use were excluded. The diagnosis of schizophrenia was ascertained on the Structured Clinical Interview for Diagnostic and Statistical Manual of Mental Disorders, fourth edition (DSM-IV).^1^ This study was approved by the National Healthcare Group’s Domain Specific Review Board. Written informed consent was obtained from all study subjects.

CATIE cohort

The final analysis included 412 schizophrenia individuals of European ancestry (n_TD_ = 62, n_Non-TD_ = 350) and 214 schizophrenia individuals of African-American ancestry (n_TD_ = 30, n_Non-TD_ = 184). Prior to quality control procedures, genotype data was available for 414 (European ancestry) and 217 (African-American ancestry) individuals with schizophrenia, and phenotype data was available for 415 (European ancestry) and 218 (African-American ancestry) individuals with schizophrenia. Detailed description of the study has been previously reported.^2–4^ Briefly, Clinical Antipsychotic Trials of Intervention Effectiveness (CATIE) is a NIMH funded multi-phase randomized control trial comparing the efficacy of typical and atypical antipsychotics in schizophrenia patients, aged between 18 and 65 years, followed-up across 18 months. Diagnosis of schizophrenia was ascertained with the Structured Clinical Interview for DSM-IV Axis I Disorder.^1^ Individuals with a diagnosis of schizoaffective disorder, first episode schizophrenia, mental retardation, and other cognitive disorders were excluded. The cohort consisted of mixed ancestry. For this study, only individuals of European and African-American descent were analyzed. Population stratification was determined via a Principal Component Analysis (PCA) of genotypes, instead of self-reported race.

# 2. Phenotype Ascertainment: Tardive Dyskinesia

TD was assessed with the Abnormal Involuntary Movement Scale (AIMS)^5^ in all cohorts. The AIMS is a clinician rated 5-point Likert scale (0 = None, 4 = Severe) that measures involuntary movement in seven body regions (i.e., orofacial, extremities and trunk). The TD status (present or absent) was classified according to the Schooler and Kane criteria.^6^ Subjects with a rating of mild dyskinesia (score of 2) on at least two or more body parts, or a rating of moderate dyskinesia (score of 3) on any body parts, were classified as having TD. 280 cases were classified as having TD and 1126 without TD (Table 1). Baseline AIMS score was used in all cohorts.

# 3. Genome-wide Genotyping, Quality Control, and Imputation

The STCRP cohort was genotyped on the Illumina 1M Duo Beadchip, and the CATIE cohort was genotyped on the Affymetrix 500K “A” chip set (Nsp and Sty chips), and Perlegen’s custom 164K chip for additional genome coverage. Prior to quality control procedures, there were 1,199,030 single nucleotide polymorphisms (SNPs) in the STCRP East Asian dataset (STCRP-EAS), 495,172 SNPs in the CATIE European descent dataset (CATIE-EUR), and 495,172 SNPs in the CATIE African-American descent dataset (CATIE-AFR).

Standard genome-wide quality control procedures were carried out using PLINK (<https://www.cog-genomics.org/plink2>) on all three ancestries (STRCP-EAS, CATIE-EUR, CATIE-AFR).

SNPs were excluded if call rate < .98 (Supplementary Figure 1); Hardy-Weinberg equilibrium p-value < 1e-6 (Supplementary Figure 3); minor allele frequency < 0.01 (Supplementary Figure 3). Samples were excluded if call rate < 0.98 (Supplementary Table 2) and inbreeding coefficient > 0.2 (Supplementary Figure 4). We visualized the data in quality control plots and removed data based on the above cut off. Invariant markers within the GWAS datasets were excluded. We aligned SNP positions and alleles to Genome Reference Consortium Human Build 37 (GRCh37) and the 1000 genome reference panel phase 1. SNPs with mismatched alleles were removed. Ambiguous ‘A-T’ and ‘C-G’ alleles were also excluded.

After marker quality control, 816,977 SNPs remained in the STCRP-EAS, 322,177 SNPs in the CATIE-EUR, and 348,202 SNPs in the CATIE-AFR. Population stratification was determined by first generating linkage disequilibrium (LD) independent variant set, and then subjected to PCA in PLINK 1.9.

The genome wide data was pruned prior to computing genome wide covariance matrix. Pruning parameters included have a window of 100kb, with sliding window of 50kb. SNPs with r^2 < 0.2 are filtered out. Identity by state/identity by descent plots was examined to exclude related samples (i.e. first and second degree relatives). Pi-hat of 0.2 (Z_0_ > 0.625) was used to unrelated samples based on visualization of IBS/IBD scatterplots (Supplementary Figure 4).

Prior to PCA, Chromosome 8 inversion region (chr8: 7000000-15000000) and Major Histocompatibility Complex region (MHC; chr6: 25000000-35000000) were removed. Linkage disequilibrium pruning was then conducted with a threshold of 100 SNPs 50 SNPs window and r^2^ = 0.2. The pruned dataset was merged with the 1000 genome reference, and 20 principal components were then generated via PLINK, for each ancestry group. K-means clustering was conducted using SPSS IBM 23.0 to identify individuals that clustered with the current samples. The first 4 principal components were used to generate 5-10 k-means clusters, and this was repeated for each pair of the first 4 principal components (i.e. PC1-PC2; PC1-PC3; PC1-PC4; PC2-PC3; PC2-PC4; PC3-PC4). This resulted in a total of 56 cluster groups. A final cluster was conducted with the 56 cluster groups and all 20 principal components to generate a k = 100 mean cluster, across the three ancestry group. The clusters were then plotted against ancestry data from the 1000 genomes project, and those that did not cluster with the reference population and its closest genetic clusters within the dataset were removed. PCA results are reported in Supplementary Figure 5.

After sample quality control, 1847 STCRP-EAS sample, 412 CATIE-EUR, and 214 CATIE-AFR samples remained. Genotype imputation was performed using Minimac3 (MaCH), as implemented on the University of Michigan imputation server,^7^ and were imputed to the 1000 Genomes Project Phase 3 version 5 reference panel. The imputation reference set consists of 5,008 haplotypes from 26 populations, with 49,143,605 markers. Cohorts from the current study were imputed against the East Asian, European and African American reference panel. Imputation yielded 47,072,232 SNPs in the STCRP-EAS, 47,109,431 SNPS in the CATIE-EUR European, and 47,109,431 SNPS in the CATIE-AFR sample

# 4. Genome-wide Association Analysis and Meta-analysis

Association analysis was carried out on the three imputed datasets via univariate linear mixed model (LMM) as implemented in GEMMA.^8^ The LMM was used to control for the inherent genetic variation observed within the CATIE-AFR dataset. To ensure consistency in analysis, LMM was applied to all three datasets. Fixed-effect inverse variance weighted meta-analysis was carried out via METAL.^9^ Filters of rsq > 0.6 and MAF filter > 3% were applied. The stringent MAF filter was applied so as to reduce the probability of type I error that may result from the small sample sizes and statistical power to detect in the current study. Further quality control where SNPs present in only one study was excluded from the meta-analysis, leaving 6,291,020 SNPs for meta-analysis. Standard genome-wide significance (p < 5x10^-8^) was applied. As a methodological control, we also implemented GWAS association analysis using logistic regression in PLINK 1.9.^10,11^ Logistic regression analysis of TD was conducted with the first 20 principal components as covariates, generated during the QC step. Results of logistic regression were also meta-analyzed using METAL.^9^

Sign test was used to evaluate the comparability of results generated from LMM via GEMMA^8^ and logistic regression via PLINK 1.9.^10,11^ A filtered set of SNPs was selected by performing a joined clump of the LMM and logistic regression GWAS meta-analysis summary statistics (P-value threshold of 1x10^-4^ for index and clumped SNPs, r^2^>0.5, and 3000kb window). SNPs in MHC region (25-35Mb) were also removed due to LD. Sign test was then performed by evaluating the number of SNPs whose beta coefficient signs were the same between the two methodologies. Under the null hypothesis, 50% of the beta coefficient signs would be the same. This analysis was performed in R program (<https://www.r-project.org/>). 892 selected top independent SNPs were identified based on joint clumping of LMM and logistic regression results. Sign test revealed that 97.1% of the SNPs had consistent effects between LMM and logistic regression (*P* < 2.2 x 10^-6^).

# 5. Functional Annotation for GWAS

GWAS summary statistics generated from the meta-analysis were entered to Functional Mapping and Annotation of genetic association (FUMA) pipeline,^12^ which generates Manhattan and QQ plots, MAGMA gene-based analysis and other functional annotation, including ANNOVAR, preliminary eQTL lookups and chromatin interactions. P-value of 5x10^-7^ was used to identify lead SNPs, significant and sub-threshold-significant genomic loci in FUMA. Other parameters included LD threshold of lead SNPs r^2^ > 0.6; secondary p-value threshold p < 0.05; MAF > 0.01; 250kb window; and 1000 genomes phase 3 European reference panel. Regional plot was visualized with LocusZoom.^13^

Expression quantitative trait loci (eQTL) were also performed as part of FUMA pipeline, using data from GTEx version 7,^14^ blood eQTL,^15^ BIOS eQTL,^16^ BrainEAC.^17^ Bonferroni correction was applied for gene-based analysis and eQTL analysis. Chromatin interaction analysis performed as part of FUMA incorporated 14 tissue types from the Hi-C data^18^ and 111 tissue/cell of enhancers and promoters from the RoadMap Epigenomics Project.^19^

# 6. Pathway Analysis

Competitive gene-set analysis was carried out in MAGMA,^20^ using all available gene-sets extracted from MSigDB v6.1.^21^ Gene-sets were limited to pathways with 10-1000 genes so as to avoid spurious findings resulting from pathways with few or too many genes,^22^ leaving a total of 17,216 gene-sets available for analysis. Bonferroni correction was applied.

# 7. Transcriptome-wide Analysis

Transcriptome-wide analysis was conducted via MetaXcan.^23^ This approach integrates GWAS summary statistics with tissue-based expression data to identify transcripts associated with TD. Tissue-based expression prediction models, derived from GTEx Version 7,^14^ were extracted from PredictDB. All 48 regions of GTEx Version 7 region were used. Gene based transcriptomic levels are trained using elastic net models, and these predicted expression levels are then correlated with the phenotype and provides a basis for gene level association test. Whereas, typical eQTL searches focuses simply on evidence of gene expression at the gene locus that is significant beyond an expression slope of 0 (https://www.gtexportal.org/home/documentationPage), MetaXcan brings analysis one step further to incorporate gene expression levels to standard GWAS summary statistics based on logistic or linear regression. The following expression summarizes the MetaXcan approach:


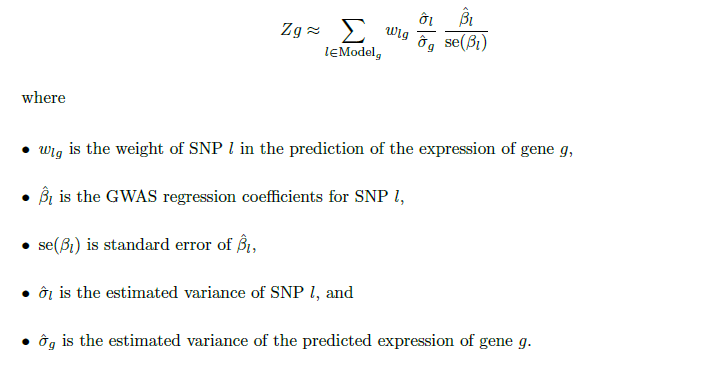


Figure adapted from Barbeira et al (2016).^23^

# 8. Fine-mapping Analysis

To identify putative causal variants in the independent loci, PAINTOR v3.1, a trans-ethnic functional fine-mapping approach was implemented.^24,25^ This approach accounts for the variability in genetic structure across ancestry by integrating LD information for each ancestry with functional annotations and GWAS summary statistics. The Gibbs sampling algorithm (-mcmc flag) was applied to identify causal variants. The 1000 genomes reference panel was used to calculate the LD for the STCRP, CATIE-EUR and CATIE-AFR cohort. PAINTOR v3.1 was implemented to a region of ±100kb from the index SNP identified in the GWAS meta-analysis. A total of 155 publicly available functional annotations relevant to ‘immune’, ‘brain’ and ‘muscle’ comprising of gene elements from GenCode,^26^ enhancer and promoter from Roadmap Epigenomics Project,^19^ and super enhancer^27^ were utilized. The annotations selected for the final fine-mapping model was conducted in two steps. First, the model for each annotation was performed separately and subsequently prioritized based on likelihood-ratio statistics.^24,25^ Second, minimally correlated top annotations (not more than five) were selected for the final model. Minimally correlated annotations were identified based on a PCA of the prioritized annotations extracted from the first step. The top annotation, based on likelihood ratio statistics, in each principal component was then selected for the final model. To obtain 99% credible SNP set, SNPs were ranked based on posterior probability, then summed till the cumulative probability reaches 99% of the total posterior probability. These were then annotated with ANNOVAR^28^ and Variant Effect Predictor.^29^ Visualization of credible SNP set is implemented in PAINTOR-CANVIS. We also carried out GCTA-COJO^30^ performing conditional analysis on the top variants from the GWAS to check if there were additional significant SNPs beyond those identified by GWAS or finemapping.

# 9. Polygenic Risk Modelling of TD with Other Diseases and Traits

To examine the degree of overlaps between the genetic architecture or more broadly the biological similarities of TD and other related neurodegenerative illnesses, polygenic risk modeling was conducted. GWAS summary statistics of neurodegenerative disorders, autoimmune conditions, psychiatric conditions, and personality traits as negative controls were downloaded from LD-HUB^31^. These included Amyotrophic Lateral Sclerosis,^32^ Alzheimer’s disease,^33^ Parkinson’s disease,^34^ Cohn’s disease,^35^ rheumatoid arthritis,^36^ schizophrenia,^37^ bipolar disorder,^38^ depression,^39^ extraversion and neuroticism.^40^ Polygenic risk score (PRS) was constructed with PRSice2^41^ for the combined sample of STCRP and CATIE by summing the risk alleles weighted by the effect size (beta or logarithm of odds ratio) as reported in the summary statistics of each investigated trait indicated above. Subsets of SNPs were selected based on fourteen P-value thresholds (P_T_ = 1x10^-5^, 1x10^-4^, .001, .01, .02, .03, .04, .05, .1, .2, .3, .4, .5, 1) from the various summary statistics. Clumping threshold of r^2^>0.1 and 500kb window was applied to address LD. SNPs in MHC region (25-35Mb) were removed to account for the complex LD in this region. Twenty PCs were also used as covariates to account for population stratification in PRS construction. Bonferroni correction of p = 0.05/10 was applied. To elucidate the shared pathways between TD and significant trait(s) identified in PRS analysis, PRS-based pathway was performed using PRSice2. The same gene-sets (MSigDB v6.1) were used, as reported above in MAGMA analysis. Ensembl release 87 (GRCh37) was used to determine gene position.^42^

# 11. Logistic Regression Modelling for Clinical and Genetic Factors Predicting TD

The sample was stratified into patients on Typical, Atypical Antipsychotic, or Both. Within each strata of antipsychotic type, we carried out logistic regression modelling, using TD case-control status as the outcome variable. The strata taking both Typical and Atypical was excluded from further analysis as sample sizes were too small, and logistic regression models appear overfitted (Nagelkerke R^2 = 1). To examine the contribution of clinical factors, and clinical with genetic factors for TD case-control status, we ran logistic regression with the null model (20 principal components + data set (SG + CATIE EUR + CATIE AFR + Sex), clinical baseline model (null model + Age of Onset, Duration of Illness, Daily CPZ equivalents) and clinical + genetic model (clinical baseline model + rs6926250, rs4237808, rs499646, rs11639774). Model level R^2 were calibrated to the liability scale, to adjust for case-control proportion and prevalence (set to 15%). To evaluate the model significance of the clinical baseline model versus clinical + genetic model we estimated chi-square difference for both models. This was repeated for each of the three antipsychotic types.

# 10. Look-up of Past Tardive Dyskinesia Studies

Variants previously associated with TD in either candidate gene or GWAS reports were extracted and compared with the current GWAS results.^43–55^ This included studies that were reviewed in Lanning et al (2016).^43^ A total of 19 variants were included in the lookup.^43–55^ A sample size weighted meta-analysis was conducted via METAL^9^ on prior variants and GWAS summary statistics from the current study. We used a p-value of 0.05 to determine prior evidence of association with TD. A p-value < 0.05 would indicate evidence of significant replication.

# 11. Supplementary Figures


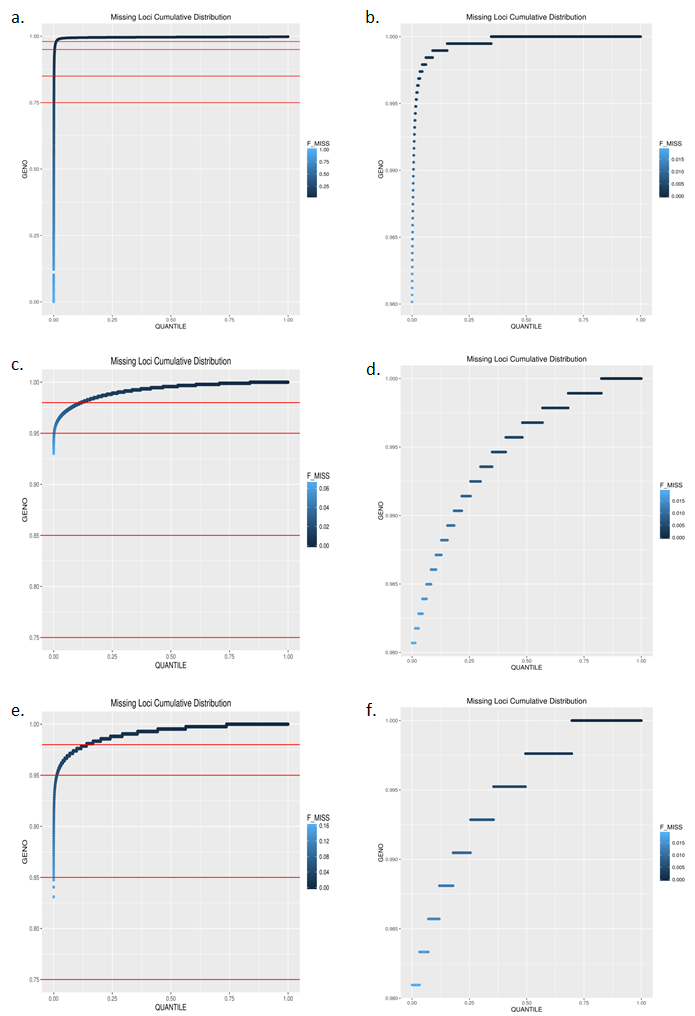


## Supplementary Figure 1. Missing SNP filters.

a. Pre-QC missing loci cumulative distribution plot for STCRP-EAS. Call rate cut-off set at 98%. Visualisation threshold are .75, .85, .95, .98. b. Post-QC missing loci cumulative distribution plot for STCRP-EAS. Call rate cut-off set at 98%. c. Pre-QC missing loci cumulative distribution plot for CATIE-EUR. Call rate cut-off set at 98%. Visualisation threshold are .75, .85, .95, .98. d. Post-QC missing loci cumulative distribution plot for CATIE-EUR. Call rate cut-off set at 98%. e. Pre-QC missing loci cumulative distribution plot for CATIE-AFR. Call rate cut-off set at 98%. Visualisation threshold are .75, .85, .95, .98. f.Post-QC missing loci cumulative distribution plot for CATIE-AFR. Call rate cut-off set at 98%. Color gradient represents rate of missing SNPs, lighter to darker gradients represent more to less missing SNPs.


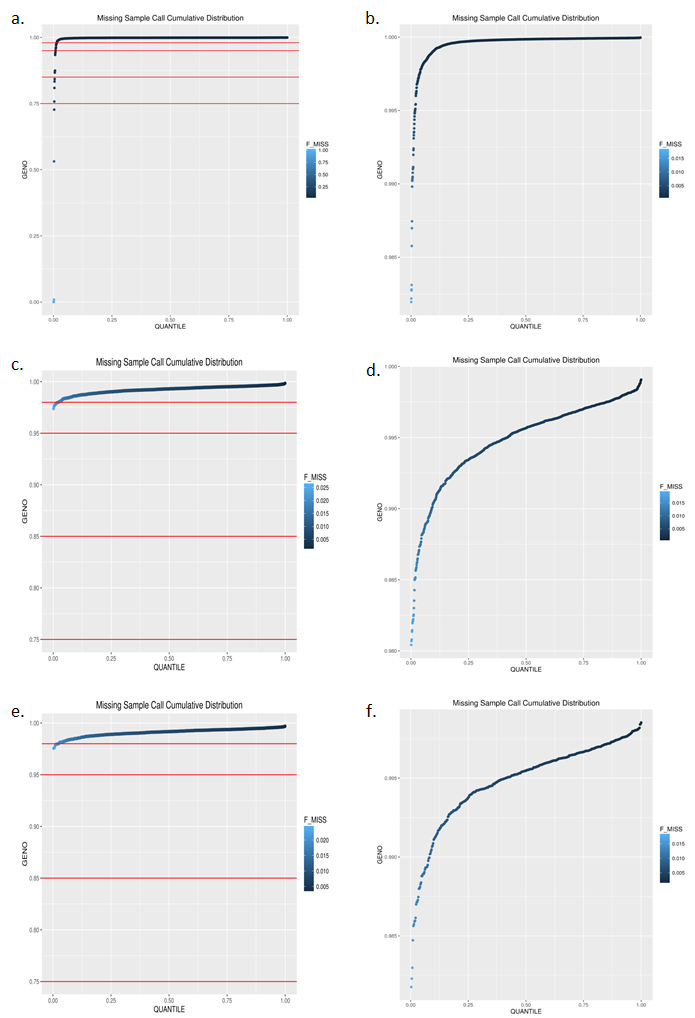


## Supplementary Figure 2. Missing individual filters.

a. Pre-QC missing sample call cumulative distribution plot for STCRP-EAS. Call rate cut-off set at 98%. Visualisation threshold are .75, .85, .95, .98. b. Post-QC missing sample call cumulative distribution plot for STCRP-EAS. Call rate cut-off set at 98%. Sample were excluded if call rate < 98%. c. Pre-QC missing sample call cumulative distribution plot for CATIE-EUR. Call rate cut-off set at 98%. Visualisation threshold are .75, .85, .95, .98. d. Post-QC missing sample call cumulative distribution plot for CATIE-EUR.. Call rate cut-off set at 98%. Sample were excluded if call rate < 98%. e. Pre-QC missing sample call cumulative distribution plot for CATIE-AFR. Call rate cut-off set at 98%. Visualisation threshold are .75, .85, .95, .98. f. Post-QC missing sample call cumulative distribution plot for CATIE-AFR. Call rate cut-off set at 98%. Sample were excluded if call rate < 98%. Color gradient represents rate of missing individuals, where lighter to darker gradient represents more to less missing rates of individuals.


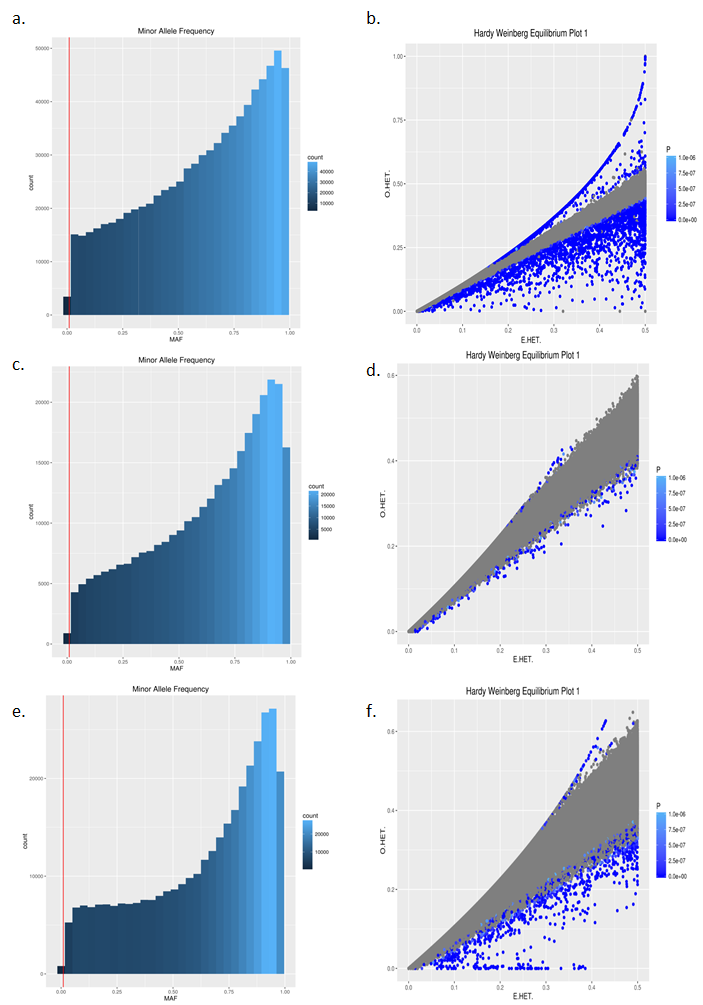


## Supplementary Figure 3. Minor Allele Frequencies and Hardy-Weinberg Equilibrium.

Distributions of minor allele frequencies are visualized for a. STCRP-EAS c. CATIE-EUR e. CATIE-AFR. Color gradient for panels a, c, and e represents number of SNPs present. Darker to lighter presents less to more SNPs. Minor allele frequencies visualized are post aligned to the 1000 genomes reference panel b37, phase 1. The red line represents the MAF < 0.01 cut-off. Hardy-Weinberg p-values are visualized for b. STCRP-EAS d. CATIE-EUR f. CATIE-AFR. SNPs within the grey polygon passed quality control, while SNPs labeled in blue were excluded. Color gradient represents p-value sizes. Lighter to darker gradients represent increasingly significant Hardy-Weinberg p-values.


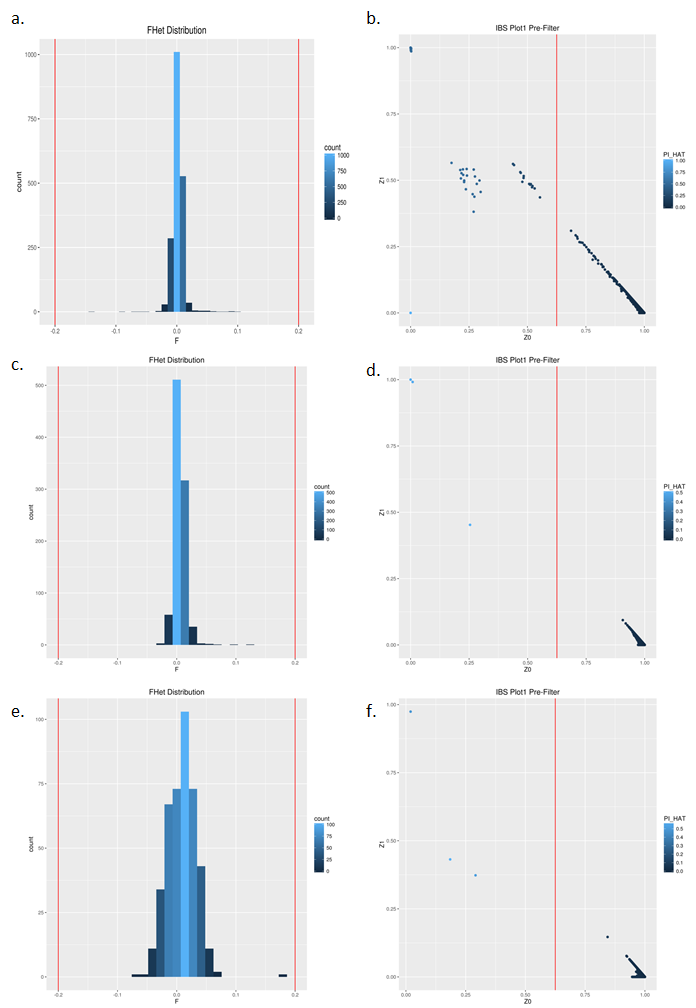


## Supplementary Figure 4. F-het coefficients and IBS/IBD filtering.

Distribution of F-het coefficients were visualized for a. STCRP-EAS c. CATIE-EUR e. CATIE-AFR post-quality control. Red lines represent F-het filter cutoffs at +/- 2.0. Color gradient represent sample frequencies, darker to lighter gradient represented frequency density of number of individuals. Identity by state/descent plot (Z0 vs Z1) for b. STCRP-EAS d. CATIE-EUR e. CATIE-AFR are visualized the three vertical panels on the right.. Z_0_ > 0.625 cut-off was used to retain unrelated individuals. Red lines represent the filter cutoff. Color gradient represented Pi-hat values. Darker to lighter gradient represent larger to smaller Pi-hat values. A Pi-hat cutoff of Pi-hat > 0.2 was jointly used with earlier Z0 cutoff to retain unrelated individuals.


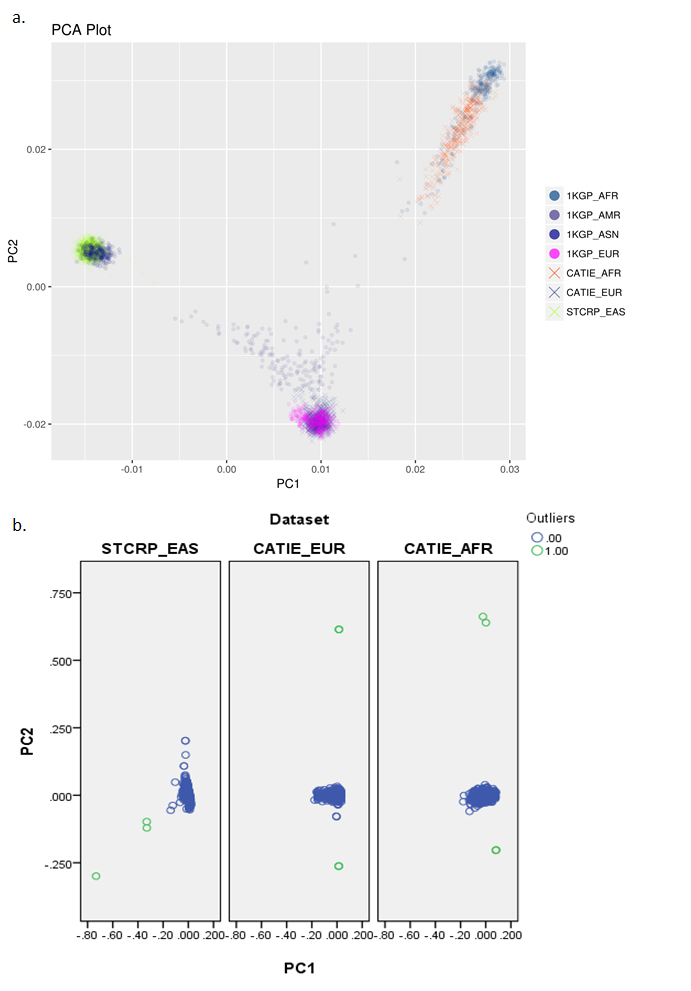


## Supplementary Figure 5. Principal components analysis for detection of population outliers.

The first two principal components “PC1” and “PC2”, x-axis and y-axis respectively were visualized in panels a and b. Panel a. Data from the 1000 genome reference panel (dots) were included in the principal components analysis and visualized along with the STCRP-EAS (Green crosses), CATIE-EUR (Pink crosses) and CATIE-AFR (Orange crosses) data. Panel b. STCRP-EAS, CATIE-EUR and CATIE-AFR scatterplots were faceted for PC1 vs PC2, k-means clustering identified potential population outliers that were highlighted in green (Outliers = 1). Individuals included in the final gwas was indicated as blue (Outliers = 0).


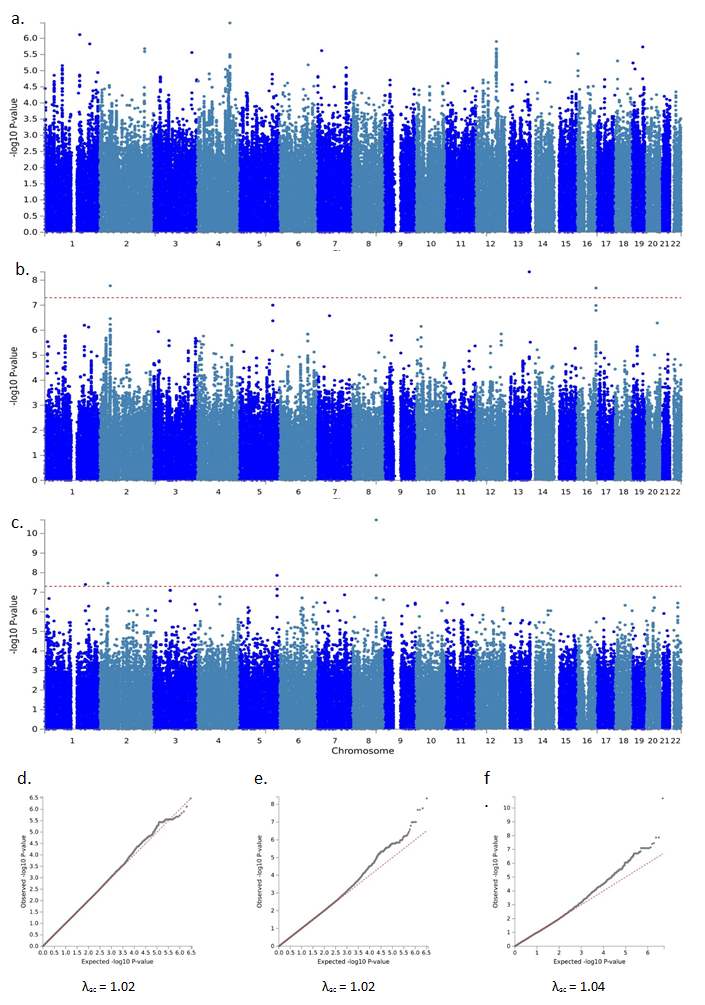


## Supplementary Figure 6. Manhattan and QQ-plots for STCRP-EAS, CATIE-EUR and CATIE-AFR.

GEMMA Linear mixed model -log10p values for a. STCRP-EAS b. CATIE-EUR and c. CATIE-AFR were visualized as manhattan plots. Dotted red lines represented significant GWAS thresholds p < 5e-8. QQ-plots and corresponding lambda values were visualized for d. STCRP-EAS e. CATIE-EUR and f. CATIE-AFR.


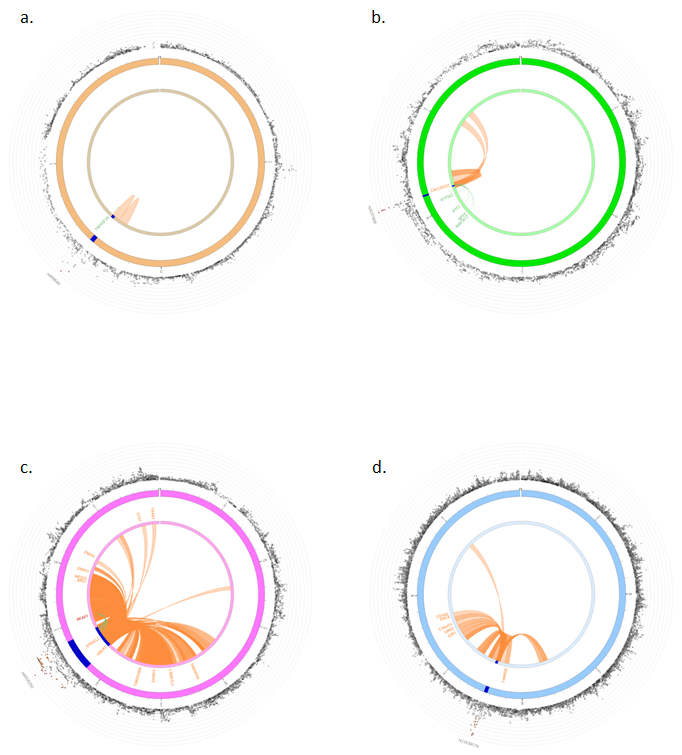


## Supplementary Figure 7. Chromatin Interaction plots for TD GWAS significant (p < 5e-8) and candidate (p < 5e-7) regions.

Orange ring represents a. Chromosome 1. Green ring represents b. Chromosome 6. Pink ring represents c. Chromosome 12 and blue ring represents d. Chromosome 16. In each region the outer ring with grey dots represent -log10p values for each SNP, red dots represent SNPs reach either GWAS significant threshold (p < 5e-8) or secondary thresholds (p < 5e-7). Dark blue region within the colored rings represent primary regions identified by GWAS p-value signals. Green lines emanating from dark blue GWAS identified regions represent presence of associated eQTL signals. Finally, orange lines emanating from dark blue GWAS identified regions represent chromatin interactions of variants with GWAS identified SNPs.


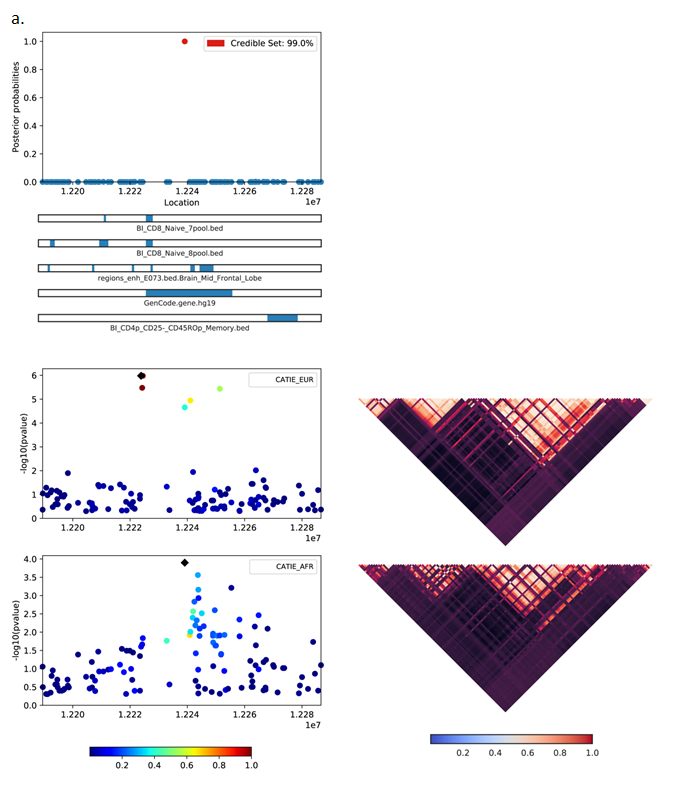


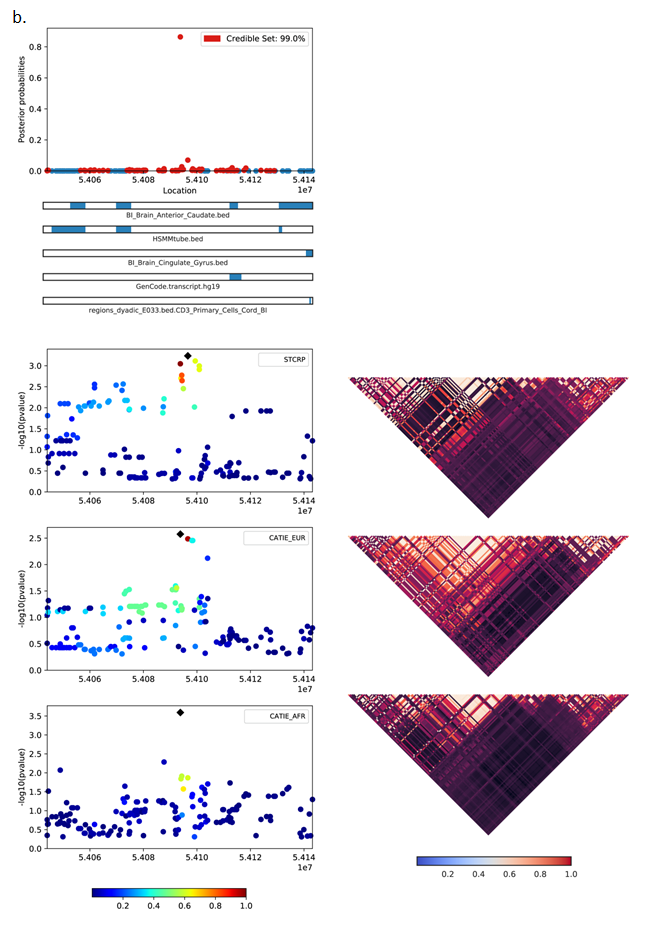


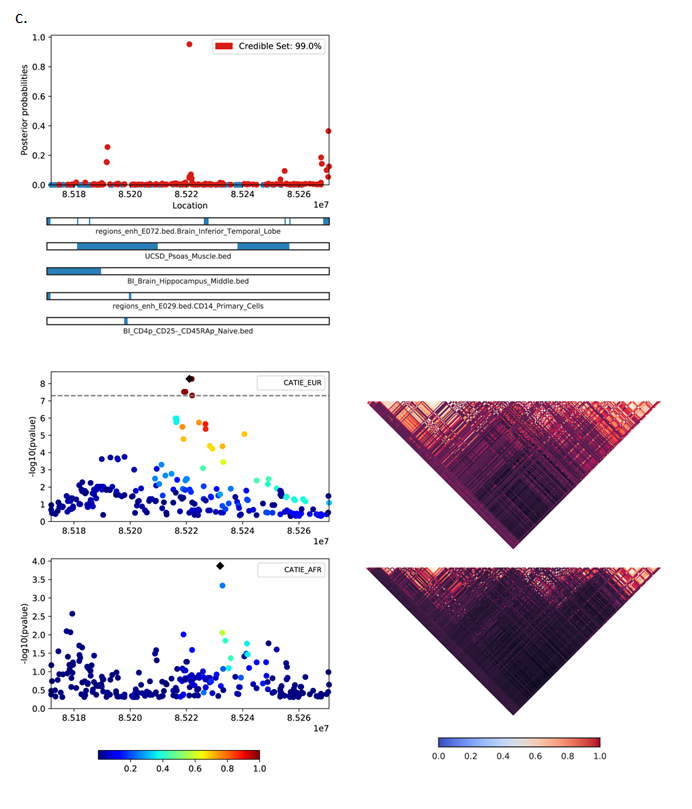


## Supplementary Figure 8. Trans-ethnic fine mapping

Visualisation of 99% credible SNP set for a. Chromosome 1, b. Chromosome 12, c. Chromosome 16. Top left panel represents the scatter plot of 99% credible SNP posterior probabilities against location of SNP, with top annotation bars. Bottom left panel represents -log10p values for each cohort. Bottom right panel represents the correlation heat-map of LD matrix for each cohort.


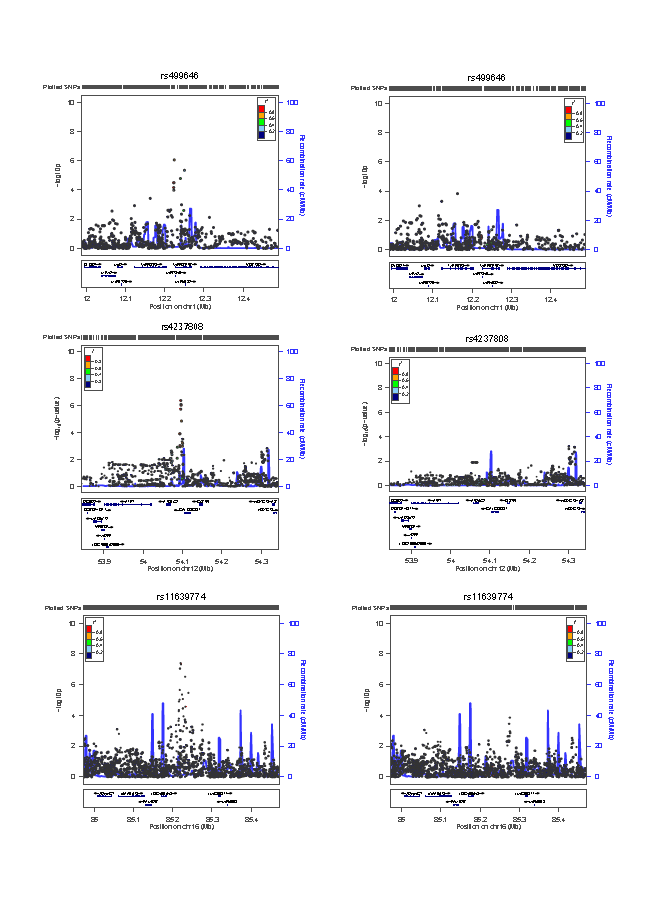


## Supplementary Figure 9. GCTA-COJO conditional analysis for top SNPs

For each of the top SNPs identified by GWAS, we performed conditional analysis to ascertain if there might be other SNPs within the region associated with Tardive Dyskinesia. Results indicated that within the stipulated recombination segment, no other SNPs were independently associated with the association outcome.

a.


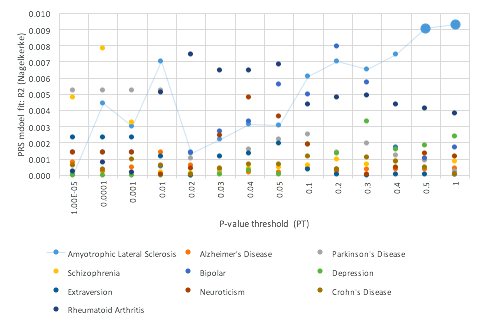


b.


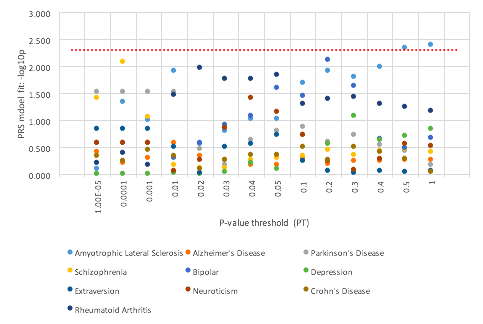


## Supplementary Figure 10. Polygenic Risk Score results.

Polygenic Risk Score prediction computed from 10 different illnesses, colored dots represent each illness. Polygenic risk score thresholds (P_T_) include 1e-5, 0.001, 0.001, 0.01, 0.02, 0.03, 0.04, 0.05, 0.1, 0.2, 0.3, 0.4, 0.5, 1. a. Nagelkerke r-square for each polygenic risk model b. -log10p values for each polygenic risk model.


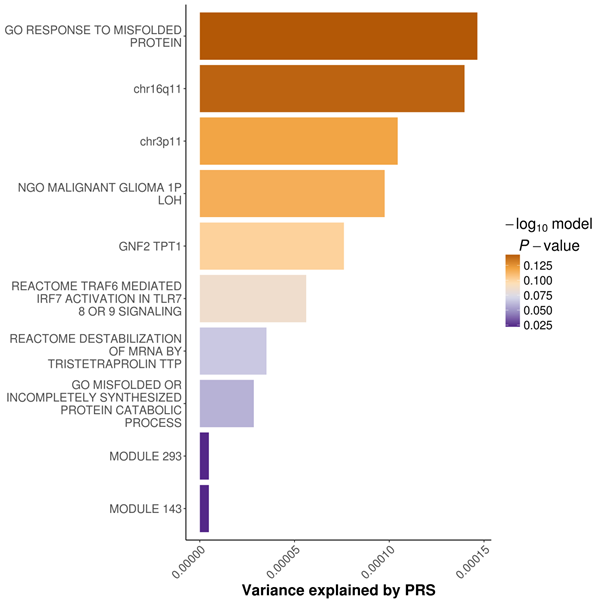


## Supplementary Figure 11. Pathway based polygenic risk score results.

Pathway based polygenic risk scores trained on Amyotrophic Lateral Sclerosis predicting TD and non-TD cases. Pathway names and functions are as follows: GO_RESPONSE_TO_MISFOLDED_PROTEIN: Any process that results in a change in state or activity of a cell or an organism (in terms of movement, secretion, enzyme production, gene expression, etc.) as a result of a misfolded protein stimulus; chr16q11: Genes in cytogenetic band chr16q11; chr3p11: Genes in cytogenetic band chr3p11; NGO_MALIGNANT_GLIOMA_1P_LOH: Proteins with reduced expression in malignant glioma cell line (A172) which bears loss of heterozygosity (LOH) in the 1p region; GNF2_TPT1: Neighborhood of TPT1 tumor protein, translationally-controlled 1 in the GNF2 expression compendium; REACTOME_TRAF6_MEDIATED_IRF7_ACTIVATION_IN_TLR7_8_OR_9_SIGNALING: Genes involved in TRAF6 mediated IRF7 activation in TLR7/8 or 9 signaling; REACTOME_DESTABILIZATION_OF_MRNA_BY_TRISTETRAPROLIN_TTP: Genes involved in Destabilization of mRNA by Tristetraprolin (TTP); GO_MISFOLDED_OR_INCOMPLETELY_SYNTHESIZED_PROTEIN_CATABOLIC_PROCESS: The chemical reactions and pathways resulting in the breakdown of misfolded or attenuated proteins.; MODULE_293: Genes in the cancer module 293; MODULE_143: Genes in the cancer module 143


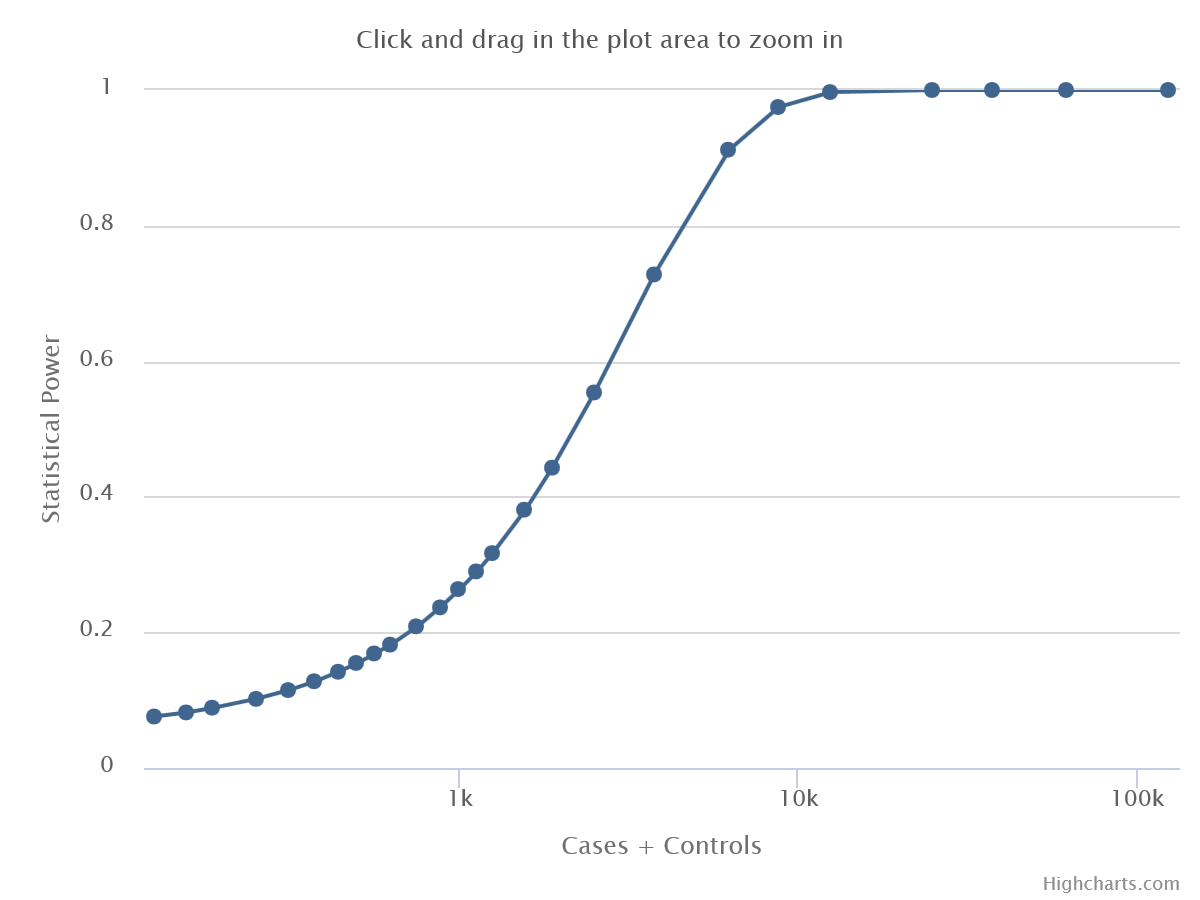


## Supplementary Figure 12. Post-Hoc GWAS power calculation.

Genetic Association Study (GAS, <http://csg.sph.umich.edu/abecasis/cats/gas_power_calculator/index.html>) Post-hoc power calculations using a 0.249 case-control ratio, and referencing on the top SNP rs11639774 (MAF = 0.168, OR = 1.163), assuming an additive model, places the variant discovery power for the current TD GWAS at 35%.

# References

1 First MB, Spitzer RL, Gibbon M, Williams JBW, Others. Structured clinical interview for DSM-IV-TR axis I disorders, research version, patient edition. SCID-I/P, 2002.

2 Lieberman JA, Stroup TS, McEvoy JP, Swartz MS, Rosenheck RA, Perkins DO *et al.* Effectiveness of antipsychotic drugs in patients with chronic schizophrenia. *N Engl J Med* 2005; **353**: 1209–1223.

3 Stroup TS, McEvoy JP, Swartz MS, Byerly MJ, Glick ID, Canive JM *et al.* The National Institute of Mental Health Clinical Antipsychotic Trials of Intervention Effectiveness (CATIE) project: schizophrenia trial design and protocol development. *Schizophr Bull* 2003; **29**: 15–31.

4 Sullivan PF, Lin D, Tzeng J-Y, van den Oord E, Perkins D, Stroup TS *et al.* Genomewide association for schizophrenia in the CATIE study: results of stage 1. *Mol Psychiatry* 2008; **13**: 570–584.

5 GUY, W. ECDEU Assessment Manual for Psychopharmacology. *NIH Guide Grants Contracts* 1976; : 534–537.

6 Schooler NR, Kane JM. Research diagnoses for tardive dyskinesia. *Arch Gen Psychiatry* 1982; **39**: 486–487.

7 Das S, Forer L, Schönherr S, Sidore C, Locke AE, Kwong A *et al.* Next-generation genotype imputation service and methods. *Nat Genet* 2016; **48**: 1284–1287.

8 Zhou X, Stephens M. Genome-wide efficient mixed-model analysis for association studies. *Nat Genet* 2012; **44**: 821–824.

9 Willer CJ, Li Y, Abecasis GR. METAL: fast and efficient meta-analysis of genomewide association scans. *Bioinformatics* 2010; **26**: 2190–2191.

10 Chang CC, Chow CC, Tellier LC, Vattikuti S, Purcell SM, Lee JJ. Second-generation PLINK: rising to the challenge of larger and richer datasets. *Gigascience* 2015; **4**: 7.

11 Purcell S, Neale B, Todd-Brown K, Thomas L, Ferreira MAR, Bender D *et al.* PLINK: a tool set for whole-genome association and population-based linkage analyses. *Am J Hum Genet* 2007; **81**: 559–575.

12 Watanabe K, Taskesen E, van Bochoven A, Posthuma D. Functional mapping and annotation of genetic associations with FUMA. *Nat Commun* 2017; **8**: 1826.

13 Pruim RJ, Welch RP, Sanna S, Teslovich TM, Chines PS, Gliedt TP *et al.* LocusZoom: regional visualization of genome-wide association scan results. *Bioinformatics* 2010; **26**: 2336–2337.

14 GTEx Consortium. Human genomics. The Genotype-Tissue Expression (GTEx) pilot analysis: multitissue gene regulation in humans. *Science* 2015; **348**: 648–660.

15 Westra H-J, Peters MJ, Esko T, Yaghootkar H, Schurmann C, Kettunen J *et al.* Systematic identification of trans eQTLs as putative drivers of known disease associations. *Nat Genet* 2013; **45**: 1238–1243.

16 Zhernakova DV, Deelen P, Vermaat M, van Iterson M, van Galen M, Arindrarto W *et al.* Identification of context-dependent expression quantitative trait loci in whole blood. *Nat Genet* 2017; **49**: 139–145.

17 Ramasamy A, Trabzuni D, Guelfi S, Varghese V, Smith C, Walker R *et al.* Genetic variability in the regulation of gene expression in ten regions of the human brain. *Nat Neurosci* 2014; **17**: 1418–1428.

18 Schmitt AD, Hu M, Jung I, Xu Z, Qiu Y, Tan CL *et al.* A Compendium of Chromatin Contact Maps Reveals Spatially Active Regions in the Human Genome. *Cell Rep* 2016; **17**: 2042–2059.

19 Roadmap Epigenomics Consortium, Kundaje A, Meuleman W, Ernst J, Bilenky M, Yen A *et al.* Integrative analysis of 111 reference human epigenomes. *Nature* 2015; **518**: 317–330.

20 de Leeuw CA, Mooij JM, Heskes T, Posthuma D. MAGMA: Generalized Gene-Set Analysis of GWAS Data. *PLoS Comput Biol* 2015; **11**: e1004219.

21 Subramanian A, Tamayo P, Mootha VK, Mukherjee S, Ebert BL, Gillette MA *et al.* Gene set enrichment analysis: a knowledge-based approach for interpreting genome-wide expression profiles. *Proc Natl Acad Sci U S A* 2005; **102**: 15545–15550.

22 Gaspar HA, Breen G. Pathways analyses of schizophrenia GWAS focusing on known and novel drug targets. bioRxiv. 2017; : 091264.

23 Barbeira A, Shah KP, Torres JM, Wheeler HE, Torstenson ES, Edwards T *et al.* MetaXcan: Summary Statistics Based Gene-Level Association Method Infers Accurate PrediXcan Results. bioRxiv. 2016; : 045260.

24 Kichaev G, Yang W-Y, Lindstrom S, Hormozdiari F, Eskin E, Price AL *et al.* Integrating Functional Data to Prioritize Causal Variants in Statistical Fine-Mapping Studies. *PLoS Genet* 2014; **10**: e1004722.

25 Kichaev G, Pasaniuc B. Leveraging Functional-Annotation Data in Trans-ethnic Fine-Mapping Studies. *Am J Hum Genet* 2015; **97**: 260–271.

26 Cunningham F, Amode MR, Barrell D, Beal K, Billis K, Brent S *et al.* Ensembl 2015. *Nucleic Acids Res* 2015; **43**: D662-9.

27 Hnisz D, Abraham BJ, Lee TI, Lau A, Saint-André V, Sigova AA *et al.* Super-Enhancers in the Control of Cell Identity and Disease. *Cell* 2013; **155**: 934–947.

28 Wang K, Li M, Hakonarson H. ANNOVAR: functional annotation of genetic variants from high-throughput sequencing data. *Nucleic Acids Res* 2010; **38**: e164.

29 McLaren W, Gil L, Hunt SE, Riat HS, Ritchie GRS, Thormann A *et al.* The Ensembl Variant Effect Predictor. *Genome Biol* 2016; **17**: 122.

30 Yang J, Lee SH, Goddard ME, Visscher PM. GCTA: a tool for genome-wide complex trait analysis. *Am J Hum Genet* 2011; **88**: 76–82.

31 Zheng J, Erzurumluoglu AM, Elsworth BL, Kemp JP, Howe L, Haycock PC *et al.* LD Hub: a centralized database and web interface to perform LD score regression that maximizes the potential of summary level GWAS data for SNP heritability and genetic correlation analysis. *Bioinformatics* 2017; **33**: 272–279.

32 van Rheenen W, Shatunov A, Dekker AM, McLaughlin RL, Diekstra FP, Pulit SL *et al.* Genome-wide association analyses identify new risk variants and the genetic architecture of amyotrophic lateral sclerosis. *Nat Genet* 2016; **48**: 1043–1048.

33 Lambert JC, Ibrahim-Verbaas CA, Harold D, Naj AC, Sims R, Bellenguez C *et al.* Meta-analysis of 74,046 individuals identifies 11 new susceptibility loci for Alzheimer’s disease. *Nat Genet* 2013; **45**: 1452–1458.

34 Simón-Sánchez J, Schulte C, Bras JM, Sharma M, Gibbs JR, Berg D *et al.* Genome-wide association study reveals genetic risk underlying Parkinson’s disease. *Nat Genet* 2009; **41**: 1308–1312.

35 Franke A, McGovern DPB, Barrett JC, Wang K, Radford-Smith GL, Ahmad T *et al.* Genome-wide meta-analysis increases to 71 the number of confirmed Crohn’s disease susceptibility loci. *Nat Genet* 2010; **42**: 1118–1125.

36 Okada Y, Wu D, Trynka G, Raj T, Terao C, Ikari K *et al.* Genetics of rheumatoid arthritis contributes to biology and drug discovery. *Nature* 2014; **506**: 376–381.

37 Schizophrenia Working Group of the Psychiatric Genomics Consortium. Biological insights from 108 schizophrenia-associated genetic loci. *Nature* 2014; **511**: 421–427.

38 Psychiatric GWAS Consortium Bipolar Disorder Working Group. Large-scale genome-wide association analysis of bipolar disorder identifies a new susceptibility locus near ODZ4. *Nat Genet* 2011; **43**: 977–983.

39 Major Depressive Disorder Working Group of the Psychiatric GWAS Consortium, Ripke S, Wray NR, Lewis CM, Hamilton SP, Weissman MM *et al.* A mega-analysis of genome-wide association studies for major depressive disorder. *Mol Psychiatry* 2013; **18**: 497–511.

40 de Moor MHM, Costa PT, Terracciano A, Krueger RF, de Geus EJC, Toshiko T *et al.* Meta-analysis of genome-wide association studies for personality. *Mol Psychiatry* 2012; **17**: 337–349.

41 Euesden J, Lewis CM, O’Reilly PF. PRSice: Polygenic Risk Score software. *Bioinformatics* 2015; **31**: 1466–1468.

42 Aken BL, Achuthan P, Akanni W, Amode MR, Bernsdorff F, Bhai J *et al.* Ensembl 2017. *Nucleic Acids Res* 2017; **45**: D635–D642.

43 Lanning RK, Zai CC, Müller DJ. Pharmacogenetics of tardive dyskinesia: an updated review of the literature. *Pharmacogenomics* 2016; **17**: 1339–1351.

44 Roberto Bakker P, Al Hadithy AFY, Amin N, van Duijn CM, van Os J, van Harten PN. Antipsychotic-Induced Movement Disorders in Long-Stay Psychiatric Patients and 45 Tag SNPs in 7 Candidate Genes: A Prospective Study. *PLoS One* 2012; **7**: e50970.

45 Tanaka S, Syu A, Ishiguro H, Inada T, Horiuchi Y, Ishikawa M *et al.* DPP6 as a candidate gene for neuroleptic-induced tardive dyskinesia. *Pharmacogenomics J* 2013; **13**: 27–34.

46 Greenbaum L, Alkelai A, Zozulinsky P, Kohn Y, Lerer B. Support for association of HSPG2 with tardive dyskinesia in Caucasian populations. *Pharmacogenomics J* 2011; **12**: 513.

47 Fedorenko OY, Loonen AJM, Lang F, Toshchakova VA, Boyarko EG, Semke AV *et al.* Association study indicates a protective role of phosphatidylinositol-4-phosphate-5-kinase against tardive dyskinesia. *Int J Neuropsychopharmacol* 2014; **18**. doi:10.1093/ijnp/pyu098.

48 Hsieh C-J, Chen Y-C, Lai M-S, Hong C-J, Chien K-L. Genetic variability in serotonin receptor and transporter genes may influence risk for tardive dyskinesia in chronic schizophrenia. *Psychiatry Res* 2011; **188**: 175–176.

49 Ivanova SA, Toshchakova VA, Filipenko ML, Fedorenko OY, Boyarko EG, Boiko AS *et al.* Cytochrome P450 1A2 co-determines neuroleptic load and may diminish tardive dyskinesia by increased inducibility. *World J Biol Psychiatry* 2015; **16**: 200–205.

50 Ivanova SA, Geers LM, Al Hadithy AFY, Pechlivanoglou P, Semke AV, Vyalova NM *et al.* Dehydroepiandrosterone sulphate as a putative protective factor against tardive dyskinesia. *Prog Neuropsychopharmacol Biol Psychiatry* 2014; **50**: 172–177.

51 Ivanova SA, Loonen AJM, Pechlivanoglou P, Freidin MB, Al Hadithy AFY, Rudikov EV *et al.* NMDA receptor genotypes associated with the vulnerability to develop dyskinesia. *Transl Psychiatry* 2012; **2**: e67.

52 Lai I-C, Mo G-H, Chen M-L, Wang Y-C, Chen J-Y, Liao D-L *et al.* Analysis of genetic variations in the dopamine D1 receptor (DRD1) gene and antipsychotics-induced tardive dyskinesia in schizophrenia. *Eur J Clin Pharmacol* 2011; **67**: 383–388.

53 Son W-Y, Lee H-J, Yoon H-K, Kang S-G, Park Y-M, Yang HJ *et al.* Gaba transporter SLC6A11 gene polymorphism associated with tardive dyskinesia. *Nord J Psychiatry* 2014; **68**: 123–128.

54 Tiwari AK, Zai CC, Likhodi O, Voineskos AN, Meltzer HY, Lieberman JA *et al.* Association study of cannabinoid receptor 1 (CNR1) gene in tardive dyskinesia. *Pharmacogenomics J* 2012; **12**: 260–266.

55 Zai CC, Tiwari AK, Mazzoco M, de Luca V, Müller DJ, Shaikh SA *et al.* Association study of the vesicular monoamine transporter gene SLC18A2 with tardive dyskinesia. *J Psychiatr Res* 2013; **47**: 1760–1765.
